# Extended Modelling of Molecular Calcium Signalling in Platelets by Combined Recurrent Neural Network and Partial Least Squares Analyses

**DOI:** 10.3390/ijms26146820

**Published:** 2025-07-16

**Authors:** Chukiat Tantiwong, Hilaire Yam Fung Cheung, Joanne L. Dunster, Jonathan M. Gibbins, Johan W. M. Heemskerk, Rachel Cavill

**Affiliations:** 1 Institute for Cardiovascular and Metabolic Research (ICMR), School of Biological Sciences, University of Reading, Whiteknights, Reading RG6 6AS, UK; chukiat.tantiwong@gmail.com (C.T.); j.m.gibbins@reading.ac.uk (J.M.G.); 2 Department of Biochemistry, Maastricht University, P.O. Box 616, 6200 MD Maastricht, The Netherlands; 3 Institute of Cardiovascular Sciences, College of Medical and Dental Sciences, University of Birmingham, Edgbaston, Birmingham B15 2TT, UK; 4 Synapse Research Institute Maastricht, Kon. Emmaplein 7, 6217 KD Maastricht, The Netherlands; 5 Department of Data Science and Knowledge Engineering, Maastricht University, P.O. Box 616, 6200 MD Maastricht, The Netherlands

**Keywords:** calcium signalling, collagen, neuronal network, platelets, thrombin

## Abstract

Platelets play critical roles in haemostasis and thrombosis. The platelet activation process is driven by agonist-induced rises in cytosolic [Ca^2+^]_i_, where the patterns of Ca^2+^ responses are still incompletely understood. In this study, we developed a number of techniques to model the [Ca^2+^]_i_ curves of platelets from a single blood donor. Fura-2-loaded platelets were quasi-simultaneously stimulated with various agonists, i.e., thrombin, collagen, or CRP, in the presence or absence of extracellular Ca^2+^ entry, secondary mediator effects, or Ca^2+^ reuptake into intracellular stores. To understand the calibrated time curves of [Ca^2+^]_i_ rises, we developed two non-linear models, a multilayer perceptron (MLP) network and an autoregressive network with exogenous inputs (NARX). The trained networks accurately predicted the [Ca^2+^]_i_ curves for combinations of agonists and inhibitors, with the NARX model achieving an R^2^ of 0.64 for the trend prediction of unforeseen data. In addition, we used the same dataset for the construction of a partial least square (PLS) linear regression model, which estimated the explained variance of each input. The NARX model demonstrated that good fits could be obtained for the nanomolar [Ca^2+^]_i_ curves modelled, whereas the PLS model gave useful interpretable information on the importance of each variable. These modelling results can be used for the development of novel platelet [Ca^2+^]_i_-inhibiting drugs, such as the drug 2-aminomethyl diphenylborinate, blocking Ca^2+^ entry in platelets, or for the evaluation of general platelet signalling defects in patients with a bleeding disorder.

## 1. Introduction

Platelets, derived from megakaryocytes, contribute to haemostasis, thrombosis, and thrombo-inflammation via receptor-induced signalling responses [1,2,3]. Physiologically important receptors are the protease-activated receptors (PAR1/4) for thrombin, the purinergic receptors (P2Y_1/12_) for ADP, which signal as G-protein coupled receptors (GPCRs), and the glycoprotein VI (GPVI) receptor for collagen, acting as a protein tyrosine kinase-linked receptor (TKLR) [4]. Since the activation and aggregation of platelets frequently drive arterial thrombotic complications [5], which are prominent causes of death worldwide [6], a clear understanding of the activation process is a must.

In platelets stimulated via GPCRs or TKLRs, rises in cytosolic [Ca^2+^]_i_ are a common initial event, contributing to essentially all platelet functions [7,8]. The receptor-induced mobilisation of Ca^2+^ from intracellular stores in the endoplasmic reticulum (or dense tubular system) proceeds via inositol 1,4,5-trisphosphate receptors (IP_3_Rs), while sarcoplasmic/endoplasmic reticulum Ca^2+^-ATPases (SERCAs) are responsible for the back pumping of Ca^2+^ into the stores (Figure S1) [7,8]. The IP_3_R channels are operated via IP_3_, which is produced as a result of activation of the GPCRs for thrombin [9] and ADP [10] and upon the activation of GPVI by collagen or collagen-related peptide (CRP) [8].

In the process of store-operated Ca^2+^ entry (SOCE), Ca^2+^ store depletion is coupled to entry of Ca^2+^ from the extracellular medium via Orai1 channels, which open upon interaction with the Ca^2+^ sensor STIM1 (stromal interaction molecule 1) in the endoplasmic reticulum membrane [7]. The back pumping of Ca^2+^ over the plasma membrane occurs via plasma membrane Ca^2+^-ATPases (PMCAs). Furthermore, primary agonists such as thrombin and CRP stimulate the release of autocrine agents, which enhance the Ca^2+^ signalling process. Particularly relevant are the autocrine agents thromboxane A_2_ (TxA_2_) and ADP, both of which stimulate IP_3_ production via GPCRs [11]. Another paracrine-dependent Ca^2+^ entry mechanism is provided by ATP, which activates P2X_1_ channels that specifically mediate Ca^2+^ entry [12].

Several pharmacological inhibitors are known to interfere with platelet Ca^2+^ responses. The entry of Ca^2+^ from blood plasma is prevented by the Ca^2+^ chelator EGTA. The back pumping of Ca^2+^ from cytosol to intracellular stores is inhibited by the compound thapsigargin, which accordingly potentiates Orai1-STIM1-dependent entry [7]. The effects of autocrine agents are suppressed by the addition of apyrase (degrading ATP and ADP) and indomethacin (blocking TxA_2_ formation). Figure S1 illustrates the actions of these platelet receptors, ligands, inhibitors, and channels relevant for the present study.

The high complexity of Ca^2+^-related signalling in platelets has led to the development of mathematical models, aiming to better understand the process and identify therapeutic targets. Authors have combined the Ca^2+^ fluxes in various platelet compartments into one model based on ordinary differential equations (ODEs) [13]. Even though this system did not include ligand–receptor interactions, it consisted of 34 entities, 35 interactions, and 86 parameters, thus reflecting the complexity of the Ca^2+^ signalling process. An alternative approach presented by Chatterjee and Diamond [14] was to create a neural network model that was trained from the Ca^2+^ response patterns to specific agonists. This neural network, acting as a black box, was able to predict synergistic effects on the Ca^2+^ responses of up to six agonists. A trade-off of the network model was that all the parameters needed to be trained, and, hence, required extensive experimental data. Another limitation of the neural network approach was that it did not predict the contribution of each Ca^2+^ channel and pump to the overall cytosolic [Ca^2+^]_i_ level.

In the present study, we constructed computational models to predict the magnitude and shape of the [Ca^2+^]_i_ time curves in platelets in response to collagen, thrombin, and CRP for a given set of experimental conditions in the absence or presence of known inhibitors. We first built two neural network models to predict agonist and inhibitor effects on the [Ca^2+^]_i_ curves. We then used partial least square (PLS) regression analysis to better understand how specific curve variables contributed to the obtained response. To exclude inter-individual variation, we used a coherent set of Ca^2+^ response curves taken from the platelets of one healthy subject, checked to be representative for five healthy subjects.

## 2. Results

### 2.1. Comparing Multiple Agonist-Induced Platelet [Ca^2+^]_i_ Curves

**Figure 1:**
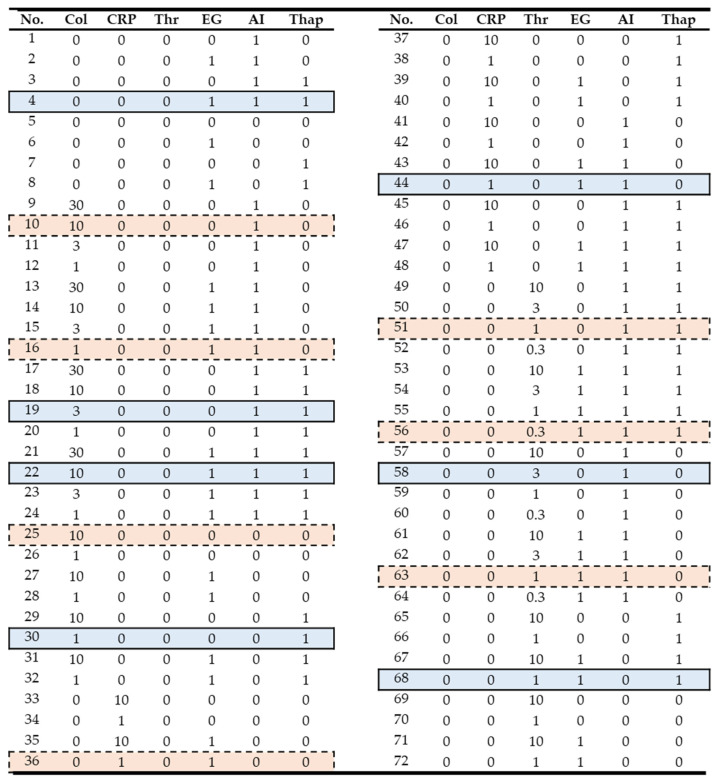
Assignment matrix of variables of 72 numbered experimental conditions. Calibrated curves of Fura-2-loaded platelets from one donor, representative for 5 donors, were used as input data. The conditions highlighted in blue (solid borders) were used as validation set, and those in red (dashed borders) were used as test set. *Abbreviations:* Col, collagen (μg/mL); CRP, collagen-related peptide (μg/mL); Thr, thrombin (nM); EG, EGTA: 0.1 mM if assigned to 1 or 1 mM CaCl_2_ if assigned to 0; AI, apyrase (0.1 U/mL) plus indomethacin (20 μM) if assigned to 1; Thap, thapsigargin (1 μM) if assigned to 1.

Using a high-throughput method described before [15], Fura-2-loaded platelets from a representative healthy donor were incubated in the presence of EGTA or CaCl_2_ with or without the secondary mediator inhibitors apyrase and indomethacin (AI) and then stimulated with collagen, CRP, or thrombin. Under these various different conditions, agonist-induced rises in [Ca^2+^]_i_ were measured as nM concentrations over a time period of 540 s. By also varying the agonist concentrations, curves were obtained for over 70 different experimental conditions. By comparing analogous sets of collagen-, thrombin-, and CRP-activated [Ca^2+^]_i_ curves from five healthy subjects [15], subject 1 was taken as representative for all (Figure S2). As shown in a comparative heatmap, the overall concordance between subjects was high per condition (experiment) and between conditions of the [Ca^2+^]_i_ rises, with a mean coefficient of variation of 29%. Accordingly, for the present analysis, we used a coherent set of 72 [Ca^2+^]_i_ time curves obtained with the platelets from subject 1 (Figure 1).

Comparing the set of original traces (Figure S3), typical characteristics were observed, in addition to the expected agonist dose dependency [15]. In general, the [Ca^2+^]_i_ curves induced by the weak GPVI agonist collagen showed steady increases with lower maximal amplitudes (Exp. 9–31) when compared to the higher amplitude and often biphasic [Ca^2+^]_i_ rises induced by the strong GPVI agonist CRP (Exp. 37–48). On the other hand, the curves with the PAR1/4 agonist thrombin (Exp. 49–72) often had a transient shape, indicating high activity of the SERCA Ca^2+^ pumps. Other differences included amplitude traces up to four times higher (depending on other variables) in the presence of CaCl_2_ compared to EGTA, which can be explained by Orai1-dependent Ca^2+^ entry [15]. Furthermore, we confirmed potent [Ca^2+^]_i_ increases with CaCl_2_ and the SERCA inhibitor thapsigargin, stimulating SOCE and activating the Orai1 channels [7]. The autocrine inhibitors indomethacin and apyrase (IA), in general, lowered most of the curves.

### 2.2. Workflow of the Modelling Approaches

**Figure 2:**
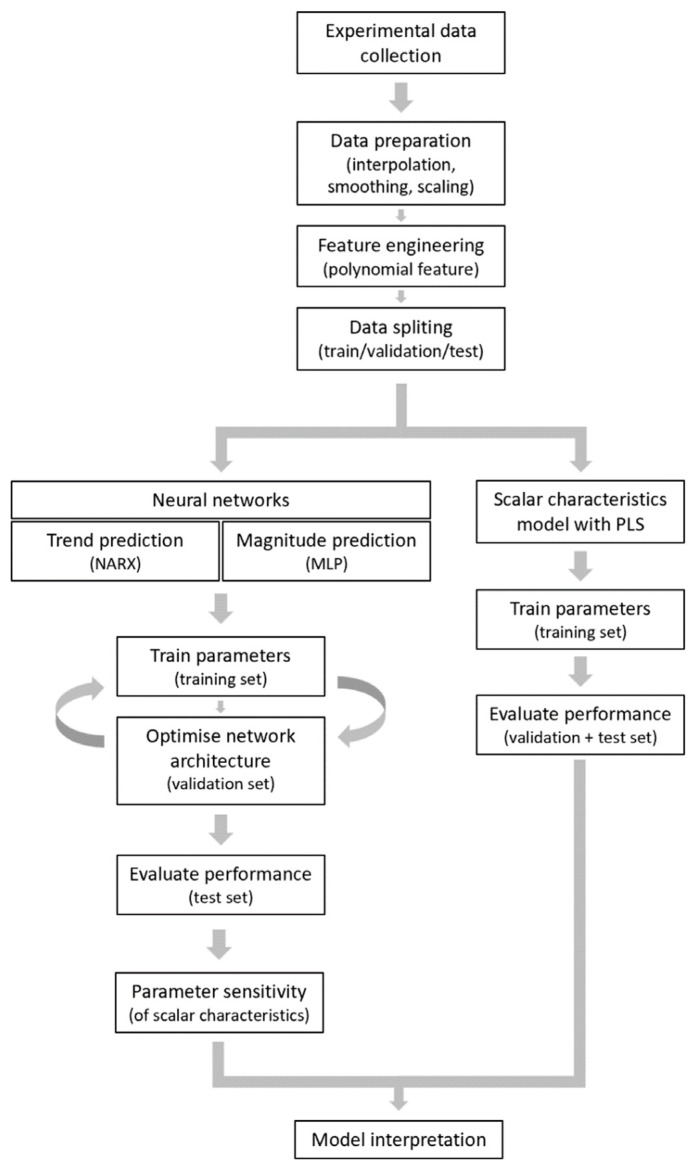
Workflow used for the data processing, neural network construction, and scalar model development. For explanation, see text.

To prepare the raw experimental data for processing, we first interpolated and smoothed the 72 curves at 1 s time intervals (Figure S4), and then *y*-axis scaled each curve in the range of 0–1 (Figure S5). The subsequent workflow (Figure 2) consisted of feature generation by combining and squaring the experimental variables (see below) and splitting the curves into training, validation, and test sets. The processed curves were then used as inputs for two types of modelling, i.e., a neural network and a PLS (partial least squares) method. Using neural network analysis, we performed an NARX (non-linear autoregressive network with exogenous input) procedure for trend prediction and an MLP (multilayer perceptron) procedure for magnitude prediction. The combined network was then tested on overall performance. Furthermore, we used PLS regression analysis to comparatively model scalar characteristics of the curves. The results from these approaches were interpreted and cross-checked with each other.

### 2.3. MLP Network for Magnitude Prediction

**Figure 3:**
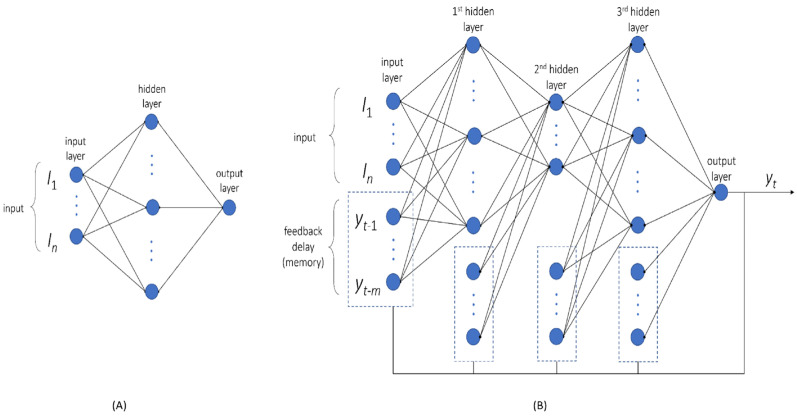
Construction of two neural networks. (**A**) Setup of MLP network as a fully connected feedforward neural network, which was used for magnitude prediction of the [Ca^2+^]_i_ time curves. (**B**) Setup of closed-loop non-linear autoregressive network with exogenous inputs (NARX), which was developed as a recurrent neural network for the trend prediction of [Ca^2+^]_i_ time curves.

We first aimed to understand how the smoothed [Ca^2+^]_i_ curves of the platelets relied on the chosen experimental conditions (CaCl_2_/EGTA, agonist, dose, AI, and thapsigargin). For this purpose, we generated a simple network able to predict the magnitude of the Ca^2+^ signal. After the training and validation of the constructed MLP network, it appeared that the best architecture had three nodes with one hidden layer (Figure 3A). We then generated plots to compare the observed data with the predictions in linear of log scale. In the plots, each data point represented an experimental and predicted magnitude value (Figure S6). The MLP parameters associated with each node are shown in Figure S7, also providing the relative weights of combined and squared parameters. The output plots indicate a reasonable fitting, especially in the log-scale setting. We concluded that the MLP approach provided suitable predictions of curve magnitudes, although this procedure did not predict curve shapes.

### 2.4. Neural NARX Network for Trend Prediction

**Figure 4:**
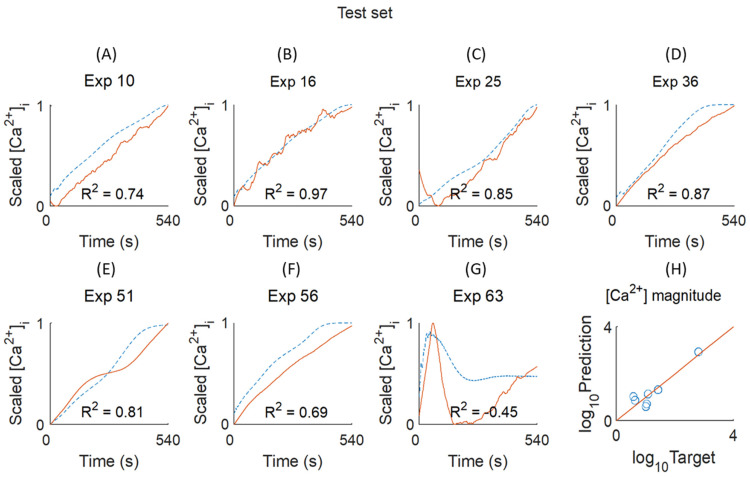
Test results of the NARX network to predict curve shapes. (**A**–**G**) Testing of trend prediction of 0–1 scaled [Ca^2+^]_i_ curves. Experiments of the test set are shown in Figure 1. Red solid lines = experimental values, blue dashed lines = predicted values. Indicated per condition are the calculated R^2^ values (a negative R^2^ indicates an explained variance worse than random). (**H**) For comparison, results from the same test set are given as obtained by the MLP network. Shown here are the target and predicted nM [Ca^2+^]_i_ levels in log scale.

To predict the shape or trend of the [Ca^2+^]_i_ curves, we applied a uniform amplitude scaling of 0–1. The developed NARX method was then used for prediction modelling of the scaled curves. For training of the NARX network, we choose 58 scaled curves (Figure S7), which resulted in the best network architecture (mean R^2^ = 0.84) with three hidden layers and 4 × 12 × 4 nodes (Figure 3B). The suitability of this network was confirmed using a validation set of seven curves (mean R^2^ = 0.71) (Figure S8). Subsequent application of a test set with seven curves resulted in a lesser fitting (R^2^ = 0.64), explained by the transiency induced by thrombin (Figure 4). In comparison, the testing of unscaled curves in the MLP network resulted in a good prediction, especially for the high-magnitude curves.

The NARX predictions provided information on the non-linear shapes of some of the [Ca^2+^]_i_ curves. Examining the R^2^ trend values, it appeared that these were negative for Exp. 58 (Figure S9) and Exp. 63 (Figure 4). This indicated an explained variance worse than random, and, hence, the inability of fitting. Furthermore, other conditions with thrombin as an agonist (Exp. 67, 70, and 72) gave a relatively low R^2^ < 0.4. This can be explained by the transiency of several thrombin-induced [Ca^2+^]_i_ rises. The apparently additive information from either procedure prompted us to integrate the neural network results of magnitude and trend prediction.

### 2.5. Combining the MLP and NARX Networks

**Figure 5:**
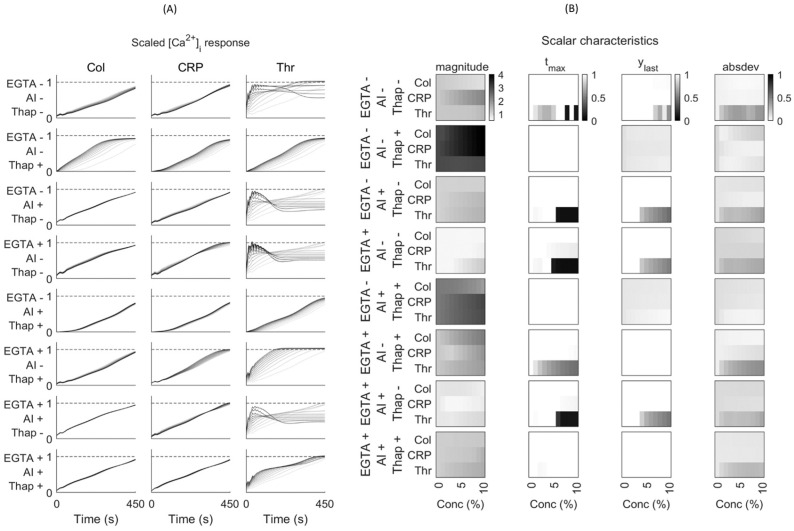
Combined effects of magnitude and trend prediction of platelet [Ca^2+^]_i_ curves at varying agonist concentrations. (**A**) Panels of prediction efficacy of scaled curves per agonist concentration. Lightest grey lines represent basal levels, while darker lines point to predicted curves in the presence of agonist at 1–10% of the maximum concentration in the training set. Columns show conditions with indicated agonists, collagen (Col), CRP, or thrombin (Thr). Rows represent different inhibitor conditions: + or − mean presence or not; from top to bottom: EGTA, apyrase plus indomethacin (AI), and thapsigargin (Thap). (**B**) Sensitivity characteristics of the scalar curves generated by MLP and NARX. Columns indicate: [Ca^2+^]_i_ level at log10 base (*magnitude*), time of [Ca^2+^]_i_ (*tmax*), final [Ca^2+^]_i_ level (*ylast*), and mean deviation from linear (*absdev*).

For a combined network prediction, we used a training set of 58 curves (Figure S10). The initial training with respect to magnitude and trend predictions was validated and tested using the remaining 14 curves (Figure S11). This combined modelling resulted in an improved outcome. We then performed one-at-a-time (OAT) factor analysis by varying the agonist concentrations from 1 to 10% of maximum at different inhibitor combinations, presenting the results as magnitude curves (Figure 5A) and heatmaps of scalar characteristics (Figure 5B).

The OAT prediction in Figure 5A indicated that the magnitude of the ‘no inhibitor’ [0 0 0] condition was more changed with the thrombin concentration when compared to collagen or CRP. The presence of EGTA reduced the magnitude prediction mostly with collagen and CRP. The thrombin concentration showed the highest magnitude effects in both the absence or presence of inhibitors. Furthermore, thapsigargin increased the overall magnitude effect at different agonist concentrations, particularly in the absence of EGTA (i.e., with CaCl2). Overall, this OAT analysis pointed to a high sensitivity of the [Ca^2+^]_i_ curves in the order of thrombin > CRP > collagen.

**Figure 6:**
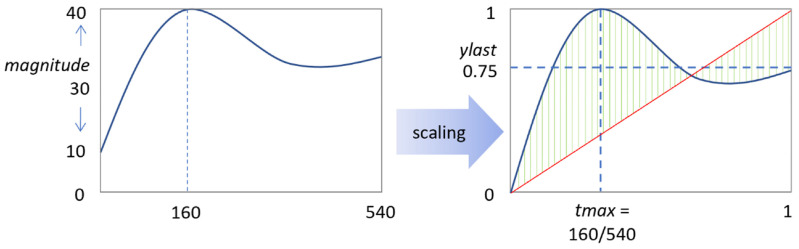
Scalar characteristic of the [Ca^2+^]_i_ time curves. Scaling resulted in the following parameters: *magnitude* (nM) points to the maximal minus minimal value of a curve; *tmax* refers to the time point where the maximal value is reached, scaled by time range (540 s). The parameter *ylast* indicates the end value, scaled according to the magnitude; *absdev* represents the mean deviation of the time curve from linear (red line). The deviation from this line (green) was calculated per time point, in which *absdev* means the average of all deviations.

Regarding the scalar curves, we compared three indications for non-linearity, i.e., *tmax*, *ylast*, and *absdev* (Figure 6). The [Ca^2+^]_i_ peak time (*tmax*) provided information on early curve saturation (<540 s). The parameter *ylast* indicated curve transiency when <1, whilst *absdev* informed on the extent of non-linearity. The heatmaps in Figure 5B show similar trends for *ylast* and *tmax*, particularly for thrombin in the absence of thapsigargin. Thus, the prediction indicated that the non-linear curve pattern with thrombin extended to higher agonist concentrations. This transiency was not seen for collagen or CRP. The analysis of *absdev* showed that most of the thrombin curves were non-monotonic, except for conditions in which thapsigargin and CaCl_2_ were present, i.e., resulting in more linear curves (Figure 5B). For all inhibitor conditions, the predicted *absdev* values for collagen and CRP were in the same lower range. Accordingly, the scaled curve characteristics informed on the Ca^2+^ response patterns at low agonist doses in the absence or presence of CaCl_2_, AI, or thapsigargin. Examination of the unscaled curves showed a better picture of the prediction at low agonist doses (Figure S12). The model thereby predicted that already low concentrations of thrombin (<0.2 nM), CRP (<0.2 μg/mL), or collagen (<0.5 μg/mL) produced relevant [Ca^2+^]_i_ rises, i.e., even below the doses inducing platelet aggregation.

From combining the tested MLP and NARX networks, several conclusions can be drawn. The transient [Ca^2+^]_i_ curves with thrombin required a different modelling approach than the non-transient responses obtained with other agonists. For the weak GPVI agonist collagen and the strong agonist CRP, the scaling approach showed monotonic curves, being close to linear at low agonist concentrations. Further, the combined modelling indicated additive effects of EGTA (Exp. 36) and AI (Exp. 42) for CRP, of which the former was stronger.

### 2.6. Partial Least Square (PLS) Regression Analyses

As an integrative approach, we then investigated how each of the experimental variables contributed to the scalar curve characteristics *tmax*, *ylast*, and *absdev*. For this purpose, we used PLS regression analysis as a linear model, directly assessing the impacts of all input variables.

As inputs for the PLS model, we normalised the experimental conditions to six variables, i.e., agonist type, concentration, EGTA/CaCl_2_, AI, and thapsigargin, which all varied from zero (none) to one (maximum). This resulted in a six-component model. As indicated in Figure S13, only the first two components contributed to the variance of the target. We then fitted the two-component PLS regression for the curves of *magnitude*, *tmax*, *ylast*, and *absdev* using the training set of 58 experimental conditions, while keeping the remaining 14 conditions (previous validation and test sets) as test set of the PLS model. Regression analysis was then used to predict the scalar characteristics of the test set, which overall showed a good or underestimated fit, but also gave errors for the training and test sets, which pointed to partial overfitting (Figure S14).

**Figure 7:**
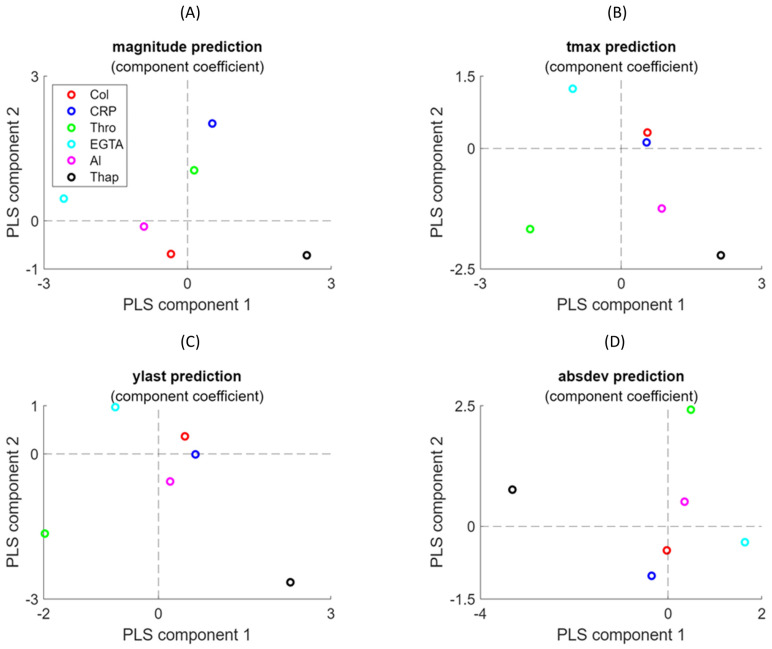
Loading coefficients of experimental variables in the PLS regression analysis. PLS regression analysis was performed for the prediction of curve *magnitude* (**A**), *tmax* (**B**), *ylast* (**C**), and *absdev* (**D**). Colours indicate contributions per variable. Plots show for two principal components the contribution of six experimental variables (collagen dose, CRP dose, thrombin dose, EGTA/CaCl_2_, AI, thapsigargin).

It appeared that the first PLS component of the *magnitude* prediction had a negative loading in the presence of EGTA and/or AI (Figure 7A), in agreement with the lower levels of [Ca^2+^]_i_ reached. On the other hand, the presence of thapsigargin resulted in a highly positive loading, linked to the increased [Ca^2+^]_i_ levels. These opposite loading coefficients reflect that the presence of EGTA prevented the entry of extracellular Ca^2+^, whereas thapsigargin increased this process by inhibiting the SERCA-type Ca^2+^ pumps controlling the STIM1-Orai1 Ca^2+^ entry pathway [15]. The agonists CRP > thrombin had strong positive predictions in component 2, indicating that these variables differed from the inhibitor effects. Furthermore, the *ylast* and *tmax* predictions showed opposite loadings in component 1 for thrombin (negative) and thapsigargin (positive) (Figure 7B,C). This reflects the transient, non-linear Ca^2+^ responses observed with thrombin in comparison to the continuously rising curves with thapsigargin. Regarding the *absdev* prediction, the thrombin condition showed a particularly high positive weight in component 2, in contrast to the negative loading in component 1 for thapsigargin (Figure 7D), also as a consequence of the different curve shapes. Accordingly, the four PLS regression models provided valuable information on the relations between curve sizes and shapes across conditions.

For validation of the combined model, based on magnitude, trend, and scalar predictions of the characteristics of agonist-induced [Ca^2+^]_i_ time curves, we evaluated the effects of a drug, 2-aminomethyl diphenylborinate (2APB), previously identified as a potent inhibitor of the STIM1-Orai1 Ca^2+^ entry pathway [15]. For this purpose, we generated 16 sets of curves with the variables collagen, thrombin, CRP, thapsigargin, and EGTA/CaCl_2_ both in the presence and absence of 2APB. For convenient and logistic reasons, we used only the high agonist concentrations. The raw curves, representative for platelets from three subjects, are provided in Figure S15A. The application of PLS regression analyses using this 16-fold dataset provided interesting insights into platelet Ca^2+^ signalling for all four curve characteristics, including *magnitude*, *tmax*, *ylast*, and *absdev*, supporting the known action mechanism of the drug. Examining the first two PLS components, the opposite loadings of EGTA and thapsigargin were retained in the magnitude and tmax predictions, in both the absence and presence of the drug (Figure S15B(a,b)). The transiency of the traces with thrombin again appeared as separate dots in the *ylast* and *absdev* predictions (Figure S15B(c,d)). Importantly, the PLS linear regression analyses also pointed to a high similarity of the curve profiles in the absence or presence of the drug 2APB, showing highly similar loadings for all variables (Figure S15B(i,ii)). In addition, when the drug was introduced as an additional variable, the loadings in components 1–2 for “Drug” and “EGTA” were close to each other (Figure S15B(iii)). This indicated that, in general, the drug 2APB did not affect the overall curve shapes, but approached the conditions with EGTA, hence confirming its action mechanism as a Ca^2+^ entry blocker regardless of the presence of agonist or other inhibitor.

## 3. Discussion

The combined modelling approaches presented here introduce a new way to predict the response size and pattern of agonist-induced platelet Ca^2+^ responses under a great variety of conditions. The constructed MLP and NARX neural networks were able to produce mostly correct magnitude curves for [Ca^2+^]_i_, whereas the modelling by PLS regression captured the characteristic curve shapes. Our work thereby adds to the idea of a platelet Ca^2+^ calculator introduced by Diamond and colleagues [14], in that, now, curve patterns can also be predicted without mathematical modelling. However, we did not consider the synergistic effects of agonist combinations such as those presented in that study.

It is important to note that, while the present machine learning techniques were able to fit most of the input data, the obtained output did not give a direct biological interpretation. This is in contrast to modelling approaches based on biological concepts, such as enzyme and receptor reaction rates in ODE-based kinetic models. However, the latter approaches cannot easily capture the complex interactions between signalling steps, for instance due to combinations of agonists and inhibitors.

Both the NARX network and PLS regression modelling yielded useful results for understanding the variation in [Ca^2+^]_i_ curves. The magnitude differences between curves in the presence of EGTA or CaCl_2_ (due to Ca^2+^ entry into the platelets) were well captured by the MLP and PLS regression models. The prediction results—i.e., sensitivity for MLP and components 1/2 for PLS—were well interpretable for this variable. On the other hand, NARX outperformed in capturing some curve variables. Thus, the subtle curve magnitude and shape effects (*tmax* and *absdev*) induced by thapsigargin were captured by NARX, but not by PLS regression. This illustrates that neural networks such as NARX can easily handle non-linear effects due to their complex activation functions, whereas PLS relies on linear regression analysis.

A specific limitation encountered was the shape differences in the [Ca^2+^]_i_ curves used for training approaches, i.e., more often transient with thrombin and non-transient with CRP or collagen. Although neural networks can capture any function, they need sufficient data to train for such curve differences. In our case, a limited number of curves per agonist was available for training, which caused an imbalance in this set. One way to fix this problem is to use data augmentation, for example, by a synthetic minority oversampling technique [16].

In the present paper, we used the platelets from a single donor for training all models, which allowed for a detailed investigation of the complex Ca^2+^ signalling pathways involved. We chose this approach because [Ca^2+^]_i_ curve aspects such as magnitude and shape often vary between blood donors [14]. However, as shown in Figure S2, it was checked for the majority of curves that the chosen subject was representative for four other healthy subjects. On the other hand, the use of blood from a single donor can be seen as a limitation, because the amount of obtained platelets reduced the number of variable experimental conditions and, accordingly, the machine learning models had a limited predictive power. These models can now be used to generate hypotheses for additional experimentation and provide insights that are otherwise not obtained by traditional analytical approaches. Appropriate use is important, ensuring that the data used for training are representative, while independent data are available for validation. However, comparing the platelet responses from a large cohort of healthy donors will increase the accuracy of overall predictions, ultimately aiming to more easily identify systematic aberrations in donors with suspected platelet bleeding disorders. Conversely, the current predictions of [Ca^2+^]_i_ rises with multiple agonists offer a foundation for estimating the thresholds for platelet activation (OAT) and for testing the effects of new antithrombotic drugs, directly or indirectly targeting platelet Ca^2+^ responses (PLS). Another application could be effect prediction in patients with gain- or loss-of-function mutations in genes encoding for Ca^2+^ response modulators, such as *STIM1* and *ORAI1* [17].

A solution to this issue is the approach of transfer learning [18], in which a generic model is built for samples from various donors and then refined to obtain adjusted weights per donor. This approach has already been used to build personalised models for drug development [19]. Regardless of the approach followed, modelled analysis will be important to understand the effects of clinically relevant inhibitors of Ca^2+^ signalling pathways, such as P2X_1_ Ca^2+^ channel antagonists [20]. In this paper, we examined this for a drug blocking the clinically important STIM1-Orai1 pathway [20], namely 2APB. The PLS regression analyses performed well, capturing the curve size and shape effects of this drug and giving loadings in the models resembling the condition “EGTA”, with no Ca^2+^ entry.

Differently from the neural network models, the PLS regression analysis performed better with the available sample size. The present PLS regression analysis to predict the (scaled) [Ca^2+^]_i_ curve features would easily allow for comparisons with platelets from more donors. In work by the Diamond laboratory [14], a NARX model was generalised by fitting networks constructed from several donors and determining their average prediction. Our analysis indicates that this can be conducted more easily by PLS regression techniques.

## 4. Methodology

### 4.1. Materials

Human α-thrombin was obtained from Kordia (Leiden, The Netherlands); cross-linked collagen-related peptide (CRP-XL) from the University of Cambridge (UK); Fura-2 acetoxymethyl ester from Invitrogen (Carlsbad, CA, USA); and Pluronic F-127 from Molecular Probes (Eugene, OR, USA). Horm-type collagen was obtained from Nycomed (Hoofddorp, The Netherlands). 2-Aminomethyl diphenylborinate (2APB) came from Sigma-Aldrich (St. Louis, MO, USA). Other materials were from sources described before [21].

### 4.2. Blood Collection and Platelet Preparation

This study was approved by the Medical Ethics Committee of Maastricht University. Blood donor age and sex could not be recorded. Blood taken into 3.2% sodium citrate (Vacuette tubes, Greiner Bio-One, Alphen a/d Rijn, The Netherlands) was obtained from consenting healthy volunteers who had not taken anti-platelet medication in the previous ten days. Platelet counts were within the reference range.

Platelet-rich plasma (PRP) was obtained from citrated blood by centrifuging, after which collected platelets were washed in the presence of apyrase (1 unit/mL) and loaded with Fura-2 acetoxymethyl ester (3 µM) and Pluronic (0.4 µg/mL) at a count of 2 × 10^8^/mL for 40 min at room temperature, as described before [22]. The isolated platelets were finally resuspended at a concentration of 2 × 10^8^/mL in Hepes buffer at pH 7.45 (10 mM Hepes, 136 mM NaCl, 2.7 mM KCl, 2 mM MgCl_2_, 5.5 mM glucose, and 0.1% bovine serum albumin).

### 4.3. Calibrated Cytosolic Ca^2+^ Measurements

In the Fura-2-loaded platelets, changes in cytosolic [Ca^2+^]_i_ were measured in 96-well plates with a FlexStation 3 (Molecular Devices, San Jose, CA, USA), as previously described [22]. When desired, the platelets in the wells were pretreated with apyrase (0.1 unit/mL) plus indomethacin (20 µM), or with thapsigargin (1 µM) for 10 min. After the addition of either 0.1 mM EGTA or 1 mM CaCl_2_, the platelets were stimulated by automated pipetting with one of the following agonists: CRP (1 or 10 µg/mL), collagen (1, 3, 10, or 30 µg/mL), thrombin (0.3, 1, 3, or 10 nM), or none of these (vehicle controls). In wells per row, changes in Fura-2 fluorescence were measured quasi-simultaneously over time at 37 °C by ratiometric fluorometry, including appropriate calibrator controls for obtaining nM concentrations of [Ca^2+^]_i_ [22]. For the independent testing of pharmacological drugs known to affect SOCE, the platelets were preincubated with 2APB (30 μM), as studied and titrated before [15,23]; the agonist concentrations were maximal: CRP 10 μg/mL or thrombin 10 nM.

### 4.4. Selection of Platelet [Ca^2+^]_i_ Curves for Modelling

For the majority of experimental conditions, the Ca^2+^ responses were studied in Fura-2-loaded platelets obtained from 5 healthy donors, thus resulting in calibrated time series of nM [Ca^2+^]_i_ [15]. For the present modelling approach, a complete set of 72 time curves was taken from subject 1 and checked to be representative for those of all subjects (Figure S2). In Figure 1, the chosen experiments for model validation and testing are highlighted in blue and red, respectively, based on criteria indicated below.

### 4.5. Preparation of Input Data

The raw curves of [Ca^2+^]_i_ changes in platelets stimulated with CRP or collagen had a sampling time of 4 s, while those with thrombin had a sampling time of 2 s. To allow for direct comparisons, the raw nM values (Figure S3) were linearly resampled and interpolated to generate 1 s time steps from 0 s to 540 s. To minimise noise disturbances, the curves were smoothed with a Savitzky–Golay filter (Figure S4).

In cases where scaling was needed, the smoothed curves were subjected to a min–max scaling algorithm, giving values between 0 and 1. To scale the input conditions, experimental variables were set as [0, 1], except for the agonist concentrations, which were scaled in the range of [0, 10] (Figure 1). Herein, 0 indicated no agonist or inhibitor present.

For constructing the multilayer perceptron (MLP) network, a regression model was built using the magnitudes of all [Ca^2+^]_i_ time series. The experimental variables were taken as inputs (Figure 3A), while the mean square error was used as a cost function. This ensured a better fit for the larger values. For this purpose, we set the target (output) for the model as log-scaled values of the nM [Ca^2+^]_i_ range as log_10_(max − min). This improved the overall accuracy of log scales.

Considering that the number of total features was small with 6 experimental variables (Figure 1), we also generated polynomial features (quadratic feature combinations), which increased this number from 6 to 27. For the MLP network, the number of hidden layers was set to 1, while the number of nodes was randomly selected from 1 to 10. The network architecture options were chosen as to train only low numbers of parameters to prevent overfitting. Networks were trained 100 times, starting from random weights. As the best structure, the network with a minimal score in the cost function of the validation set was taken. Network training was performed using the Levenberg–Marquardt algorithm, containing a rectified linear unit as the activation function in each node. The modelling was conducted using Matlab R2022a and the Neural Network Toolbox.

### 4.6. Trend Prediction of NARX Network

A separate neural network was constructed to predict the trends (shapes) of smoothed and scaled [Ca^2+^]_i_ time curves. To better capture the time dynamics, we chose a non-linear autoregressive network with exogenous input (NARX) and parallel architecture [24,25], which is also known as a closed-loop neural network. For this NARX network, the model’s output *y*(*t*) was used to fit the target (i.e., the smoothed and scaled [Ca^2+^]_i_ curves). The output then generated feedback as an additional input to the network when combined with the experimental condition (Figure 3B). The mathematical expression for [Ca^2+^](*t*) is then written as follows:

(1) $$y\left(t\right)=f\left(L_{4}\times f\left(H_{3}\times y_{h}+L_{3}\times f\left(H_{2}\times y_{h}+L_{2}\times f\left(H_{1}\times y_{h}+W\times I+b_{1}\right)+b_{2}\right)+b_{3}\right)+b_{4}\right)$$

where *y*(*t*) is [Ca^2+^]_i_ over time, *I* is an input matrix of the experimental conditions, and *y_h_* is the feedback delay (history) of *y*. Furthermore, *W* and *H_n_* are the input matrix weight and feedback delay of y, respectively; *b_n_* are biases; *L_n_* are the weights of each hidden layer; and *f* is the activation (transfer) function. Note that the product of the matrix is also a matrix, meaning that the equation represents a summation of numerous parameters and functions.

For feedback delays, we chose the values at 1, 3, 6, 10, 15, 21, 28, and 36 s prior to the current value of a [Ca^2+^]_i_ time series. Hence, these feedback delays kept the information about current values, while preserving the long-term memory of the system. The initial values of the feedback delays were set to zero, as the system was assumed to be in a steady state prior to the agonist-induced activation of platelets. The use of MSE as a cost function allowed us to make predictions of the scaled min–max [Ca^2+^]_i_ time series. Scaling was performed per time series, implying that each series had the same range [0, 1]. Polynomial features were used also in this network, thus expanding the number of inputs from 6 to 27.

The neural network architecture was optimised to maximise the goodness of fit but to prevent overfitting. We used three hidden layers, with each layer’s size varying between 2 and 20 nodes (not including feedback delays). This gave approximately 7000 different architectures being trained. A randomised grid search was employed to find the best architecture. For training, the Levenberg–Marquardt algorithm was used with a hyperbolic tangent sigmoid as an activation function. Since parameter fitting in the neural network depended on a random seed, each architecture was fitted 100 times, after which the best parameters were used for comparison. The networks were built and trained in Matlab R2022a.

### 4.7. Parameter Sensitivity Analysis

To perform agonist concentration sensitivity analysis, the method of one-at-a-time (OAT) factor was applied [26]. This kept the variables fixed to the central or baseline value, while changing one variable at a time. Since effects were computed with reference to the same central point in space, this improved the comparability of the outcomes. As default, we set the conditions of EGTA or CaCl_2_, autocrine inhibitors (AI) or not, and thapsigargin or not as 1 or 0 (2^3^ = 8 combinations). Furthermore, we scaled the agonist concentration from 0 to 10% of the maximal concentrations (30 μg/mL collagen, 10 μg/mL CRP, or 10 nM thrombin). The shape of each [Ca^2+^]_i_ time curve was defined according to four scalar characteristics, namely the magnitude of the response, peak time, relative terminal level, and the mean deviation from a straight line (Figure 2).

### 4.8. Partial Least Square (PLS) Regression Analysis

Regression analysis with PLS was used as an extension of principal component analysis [27,28], which maximises the covariance between an input matrix X and output matrix Y. In this method, each component has a latent variable *t_i_*, while the linearly weighted combination of the latent variables generates the prediction of outcomes (Y matrix), as follows:

Y = C_1_*t*_1_ + C_2_*t*_2_ + …, where C_i_ = a_1i_x_1_ + a_2i_x_2_ (2)

The experimental conditions of Figure 1 were used as the X matrix and the scalar characteristics of a [Ca^2+^]_i_ time series were used as the Y matrix. The number of components in the PLS analysis was taken from the optimal variance achieved. The loading weights depended on the input variables that contributed most to the prediction. By maximising the covariance between explanatory variable X and response variable Y, the most relevant components in X were obtained for changes in Y. Stated otherwise, by examining the loading weights of a few latent variables accounting for most of the explained covariance, we could identify the experimental conditions with the most significant impact on the [Ca^2+^]_i_ time curves.

## 5. Conclusions

Of the two developed non-linear models, a multilayer perceptron (MLP) network and an autoregressive network with exogenous inputs (NARX), the trained networks accurately predicted platelet [Ca^2+^]_i_ curves in the presence of combinations of agonists and inhibitors. The NARX model achieved good results for the trend prediction of unforeseen data. Furthermore, the NARX model demonstrated good fits for the modelled calcium curves, whereas the PLS regression models gave useful interpretable information on the importance of each variable. These modelling results are suitable for the development of novel platelet [Ca^2+^]_i_-inhibiting drugs, as we demonstrated for the drug 2APB, blocking agonist-induced Ca^2+^ entry in platelets.

## Data Availability

The data are included in the manuscript as figures, tables, or Supplementary Materials.

## Supplementary Materials

The following supporting information can be downloaded at: https://www.mdpi.com/article/10.3390/ijms26146820/s1.

## References

[1] Patel S.R., Hartwig J.H., Italiano J.E. (2005). The biogenesis of platelets from megakaryocyte proplatelets. J. Clin. Investig..

[2] Chesterman C., Owe-Young R., Macpherson J., Krilis S. (1986). Substrate for endothelial prostacyclin production in the presence of platelets exposed to collagen is derived from the platelets rather than the endothelium. Blood.

[3] Davis G.E., Senger D.R. (2005). Endothelial extracellular matrix: Biosynthesis, remodelling, and functions during vascular morphogenesis and neovessel stabilisation. Circ. Res..

[4] Van der Meijden P.E., Heemskerk J.W. (2019). Platelet biology and functions: New concepts and clinical perspectives. Nat. Rev. Cardiol..

[5] Jerjes-Sánchez C. (2005). Venous and arterial thrombosis: A continuous spectrum of the same disease?. Eur. Heart J..

[6] Jackson S.P. (2011). Arterial thrombosis-insidious, unpredictable and deadly. Nat. Med..

[7] Mammadova-Bach E., Nagy M., Heemskerk J.W., Nieswandt N., Braun A. (2019). Store-operated calcium entry in blood cells in thrombo-inflammation. Cell Calcium.

[8] Versteeg H.H., Heemskerk J.W., Levi M., Reitsma P.S. (2013). New fundamentals in hemostasis. Physiol. Rev..

[9] Watson S.P., McConnell R.T., Lapetina E.G. (1984). The rapid formation of inositol phosphates in human platelets by thrombin is inhibited by prostacyclin. J. Biol. Chem..

[10] Daniel J.L., Dangelmaier C.A., Selak M., Smith J.B. (1986). ADP stimulates IP_3_ formation in human platelets. FEBS Lett..

[11] Capra V., Bäck M., Angiolillo D.J., Cattaneo M., Sakariassen K.S. (2014). Impact of vascular thromboxane prostanoid receptor activation on hemostasis, thrombosis, oxidative stress, and inflammation. J. Thromb. Haemost..

[12] Oury C., Toth-Zsamboki E., Thys C., Tytgat J., Vermylen J., Hoylaerts M.F. (2001). The ATP-gated P2X_1_ ion channel acts as a positive regulator of platelet responses to collagen. Thromb. Haemost..

[13] Dolan A.T., Diamond S.L. (2014). Systems modelling of Ca^2+^ homeostasis and mobilisation in platelets mediated by IP_3_ and store-operated Ca^2+^ entry. Biophys. J..

[14] Chatterjee M.S., Purvis J.E., Brass L.F., Diamond S.L. (2010). Pairwise agonist scanning predicts cellular signalling responses to combinatorial stimuli. Nat. Biotechnol..

[15] Cheung H.Y., Zou J., Tantiwong C., Fernández D.I., Huang J., Ahrends R., Roest M., Cavill R., Gibbins J.M., Heemskerk J.W. (2023). High-throughput assessment identifying major platelet Ca^2+^ entry pathway via tyrosine kinase-linked and G protein-coupled receptors. Cell Calcium.

[16] Chawla N.V., Bowyer K.W., Hall L.O., Kegelmeyer W.P. (2002). SMOTE: Synthetic minority over-sampling technique. J. Artific. Intell. Res..

[17] Shawer H., Norman K., Cheng C.W., Foster R., Beech D.J., Bailey M.A. (2021). ORAI1 Ca^2+^ channel as a therapeutic target in pathological vascular remodelling. Front. Cell Dev. Biol..

[18] Neyshabur B., Sedghi H., Zhang C. What is being transferred in transfer learning?. Proceedings of the 34th International Conference on Neural Information Processing Systems.

[19] Dana D., Gadhiya S.V., Surin L.G., Li D., Naaz F., Ali Q., Paka L., Yamin M.A., Narayan M., Goldberg I.D. (2018). Deep learning in drug discovery and medicine; scratching the surface. Molecules.

[20] Bennetts F.M., Mobbs J.I., Ventura S., Thal D.M. (2022). The P2X_1_ receptor as a therapeutic target. Purinergic Signal..

[21] Gilio K., Munnix I.C., Mangin P., Cosemans J.M., Feijge M.A., van der Meijden P.E., Olieslagers S., Chrzanowska-Wodnicka M.B., Lillian R., Schoenwaelder S. (2009). Non-redundant roles of phosphoinositide 3-kinase isoforms α and β in glycoprotein VI-induced platelet signalling and thrombus formation. J. Biol. Chem..

[22] Jooss N.J., De Simone I., Provenzale I., Fernández D.I., Brouns S.L., Farndale R.W., Henskens Y.M., Kuijpers M.J., ten Cate H., van der Meijden P.E. (2019). Role of platelet glycoprotein VI and tyrosine kinase Syk in thrombus formation on collagen-like surfaces. Int. J. Mol. Sci..

[23] Zou J., Zhang P., Solari F.A., Schönichen C., Provenzale I., Mattheij N.J., Kuijpers M.J., Rauch J.S., Swieringa F., Sickmann A. (2024). Suppressed ORAI1-STIM1-dependent Ca^2+^ entry by protein kinase C isoforms regulating platelet procoagulant activity. J. Biol. Chem..

[24] Xie H., Tang H., Liao Y. Time series prediction based on NARX neural networks: An advanced approach. Proceedings of the 2009 International Conference on Machine Learning and Cybernetics.

[25] Hewamalage H., Bergmeir C., Bandara K. (2021). Recurrent neural networks for time series forecasting: Current status and future directions. Int. J. Forecast..

[26] Razavi S., Gupta H.V. (2015). What do we mean by sensitivity analysis? The need for comprehensive characterisation of global sensitivity in earth and environmental systems models. Water Resourc. Res..

[27] Wold S., Sjöström M., Eriksson L. (2001). PLS-regression: A basic tool of chemometrics. Chemom. Intell. Lab. Syst..

[28] Abdi H. (2010). Partial least squares regression and projection on latent structure regression (PLS regression). Wiley Interdiscip. Rev. Comput. Stat..

