# Supplementary material for: Extended Modelling of Molecular Calcium Signalling in Platelets by Combined Recurrent Neural Network and Partial Least Squares Analyses
Source: Int J Mol Sci. 2025 Jul 16;26(14):6820. doi: 10.3390/ijms26146820 (PMC12295713; doi:10.3390/ijms26146820)
Supplement: Supplementary file 1 [file ijms-26-06820-s001.zip › ijms-3407304-supplementary.pdf]

Supplemental file IJMS

Extended Modelling of Platelet Calcium Signalling by Combined Recurrent Neural Network and Partial Least Squares Analyses

Chukiat Tantiwong[1,2], Hilaire Yam Fung Cheung[2,3], Joanne L Dunster[1], Jonathan M. Gibbins[1], Johan W. M. Heemskerk[1,4] and Rachel Cavill[5]

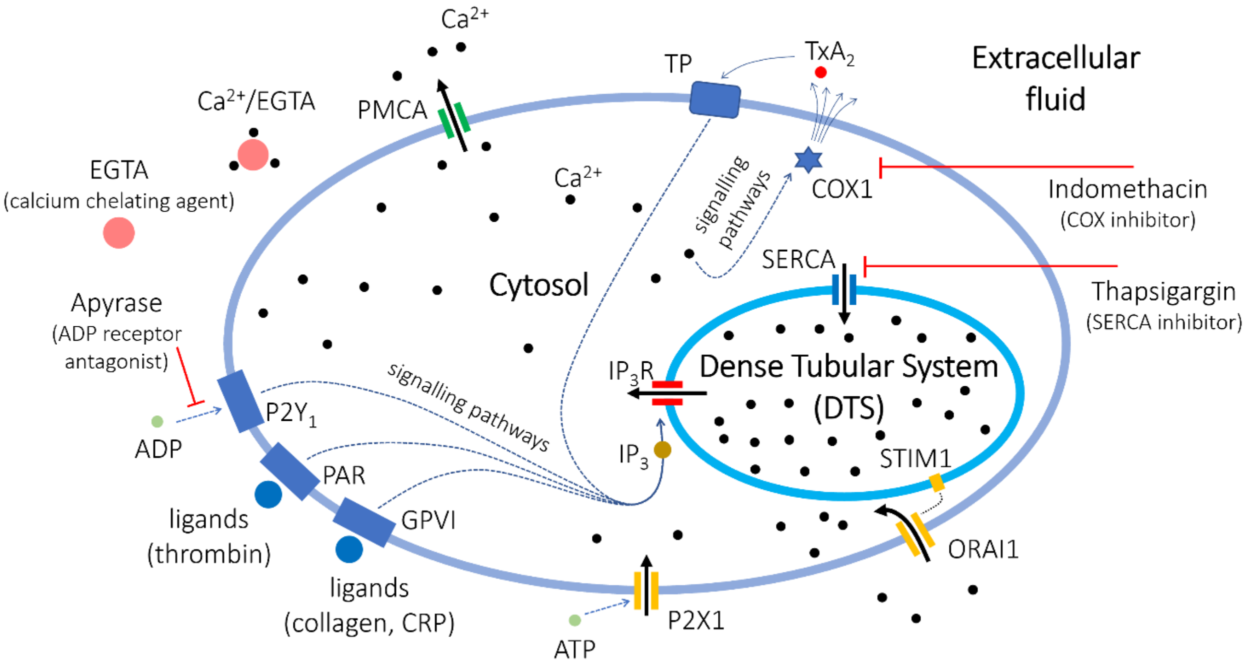

Figure S1. Overview of receptor-induced Ca^{2+} signalling mechanisms in platelets. Collagen and collagen-related peptide (CRP) activate platelet via glycoprotein GPVI (GPVI), while thrombin acts by cleaving proteinase-activated receptors (PAR). The ligands of these receptors induce the formation of inositol 1,4,5-trisphosphate (IP_3), which stimulates IP_3 receptors (IP_3R) in the membrane of the dense tubular system (DTS). This stimulation leads to discharge of Ca^{2+} from intracellular stores into the cytosol. Entry of extracellular Ca^{2+} is mediated by the Orai1 Ca^{2+} channels in the plasma membrane, which couple to STIM1 Ca^{2+} sensors in the DTS membrane. In addition, a fast and quickly desensitized entry of Ca^{2+} is mediated by ATP, activating the $P2X_1$ ion channels. Autocrine produced ADP and thromboxane A_2 (TxA_2), via their receptors, potentiate the IP_3 production. Back pumping of released Ca^{2+} out of the cytosol occurs by the SERCA Ca^{2+} ATPases in the DTS, which are inhibited by thapsigargin. Back pumping out of the cells occurs by the PMCA Ca^{2+} ATPases. The presence of extracellular $CaCl_2$ or EGTA allows or blocks Ca^{2+} entry, respectively. The formation of autocrine TxA_2 is inhibited by indomethacin, whereas autocrine ADP is degraded by apyrase (indicated in text as autocrine inhibitors, AI).

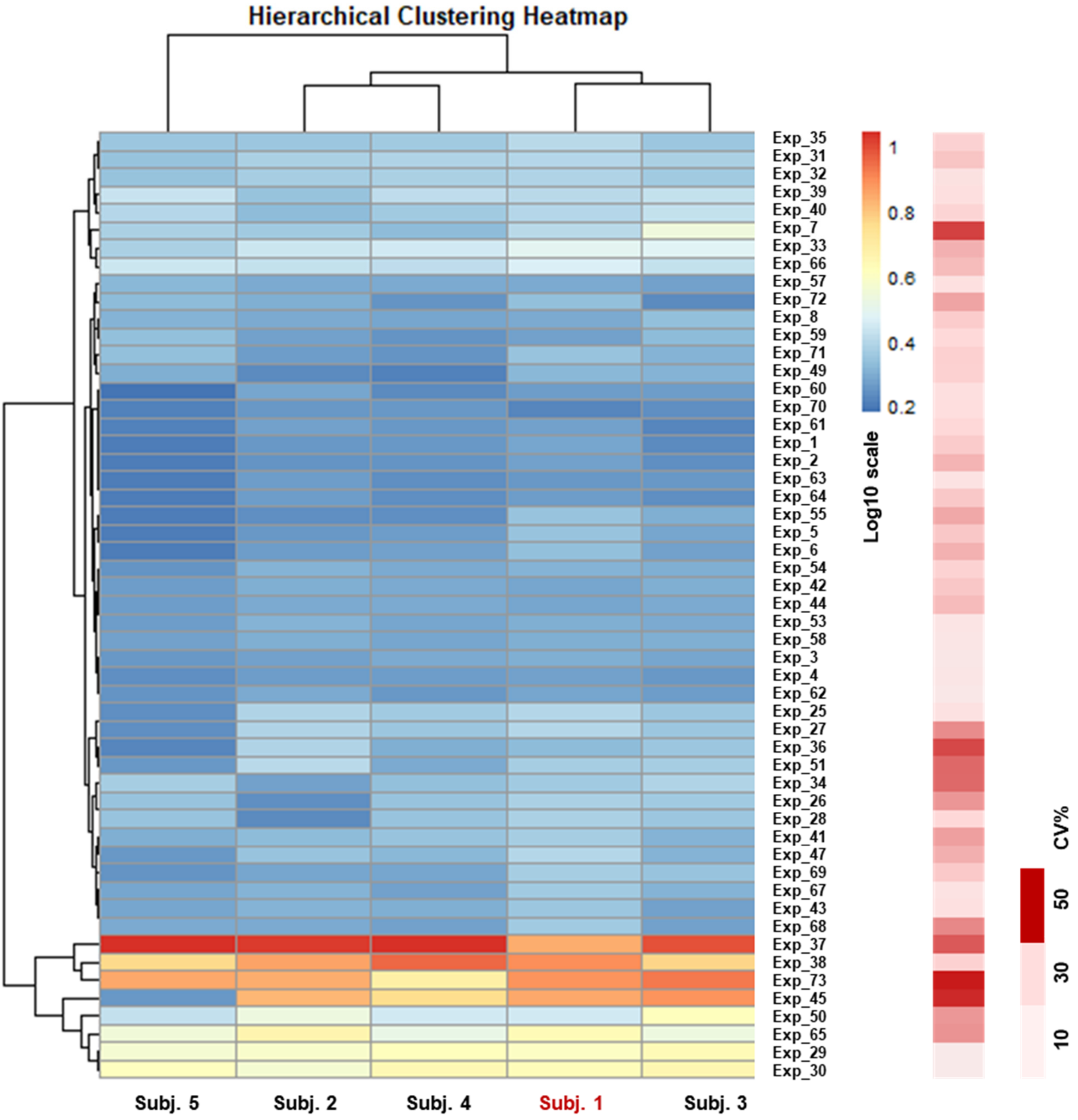

Figure S2. Concordance of agonist-induced $[Ca^{2+}]_i$ rises in platelets from five healthy subjects. Fura-2-loaded platelets from subjects 1-5 were triggered at a variety of conditions. Nanomolar rises in $[Ca^{2+}]_i$ were measured over 540 s at 37 °C, as described in the methods. Agonists were collagen, CRP or thrombin; inhibitors were EGTA, AI or thapsigargin. Experiment numbering was as in Table 1; Exp 73 refers to CRP 10 µg/mL + $CaCl_2$. Smoothened curves were analyzed for scalar characteristics: magnitude of response (peak), slope, transiency, final level, and area-under-the- curve. Obtained 7 parameters per curve were uniformly scaled 0-10 before averaging. Shown is an unsupervised clustered heatmaps of log10 scaled curve values per subject. Mean (SD) coefficient of variance of curves between subjects across all experiments was 29 ± 10% (see color legend). For magnitude curves of subject 1, see Figure S3.

Raw $[Ca^{2+}]_i(t)$ traces

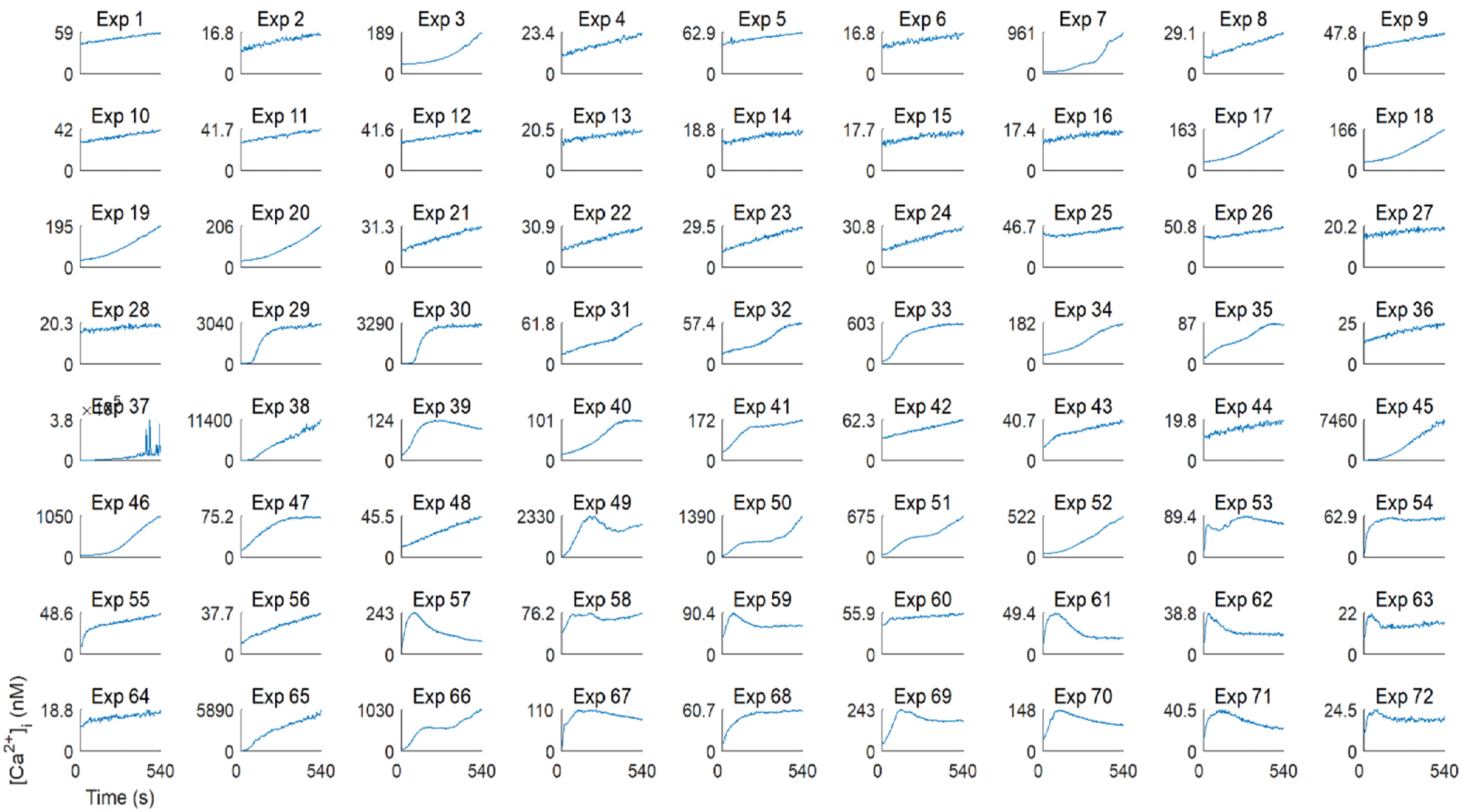

Figure S3. Raw data of agonist-induced $[Ca^{2+}]_i$ time curves of Fura-2-loaded platelets from a single donor, used for the modelling studies (subject 1). Note widely different ranges of nanomolar levels of $[Ca^{2+}]_i$ per condition. *Y*-axes represent linear ranges in nM $[Ca^{2+}]_i$. Time axes are 0 to 540 s.

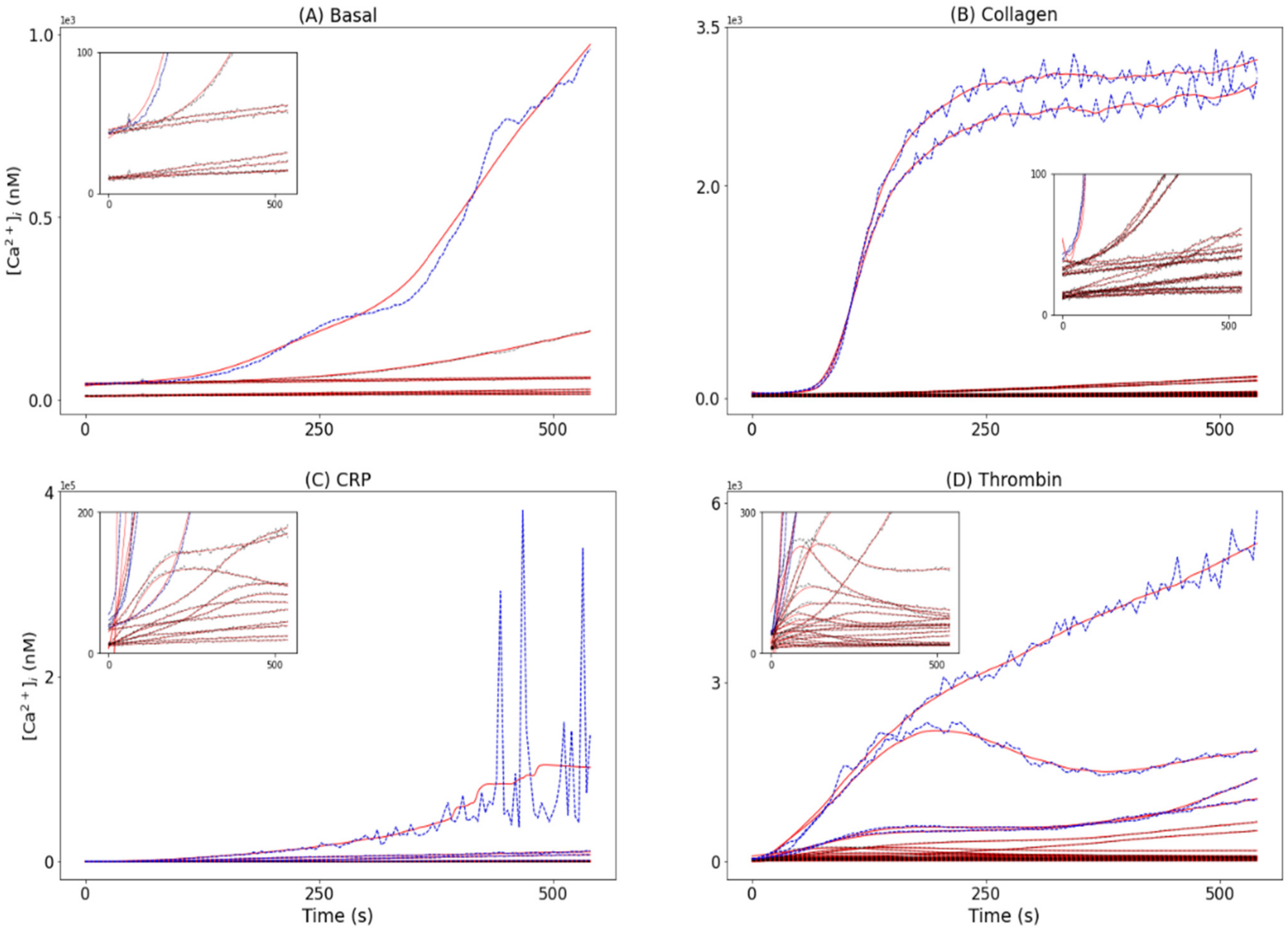

Figure S4. Raw and smoothened agonist-induced $[Ca^{2+}]_i$ curves of Fura-2-loaded platelets from a representative subject. The 72 experimental conditions (Table 1) were grouped into four panels according to the agonist used: (**A**) basal (no agonist), (**B**) collagen, (**C**) CRP, or (**D**) thrombin. Resampled and interpolated curves were smoothened with a Savitzky-Golay filter. Shown are the original curves (dash black lines) and the filtered curves (red lines). Note that highest supra-micromolar rises in $[Ca^{2+}]_i$ were obtained in the presence of thapsigargin and $CaCl_2$ (dashed blu*e lines*).

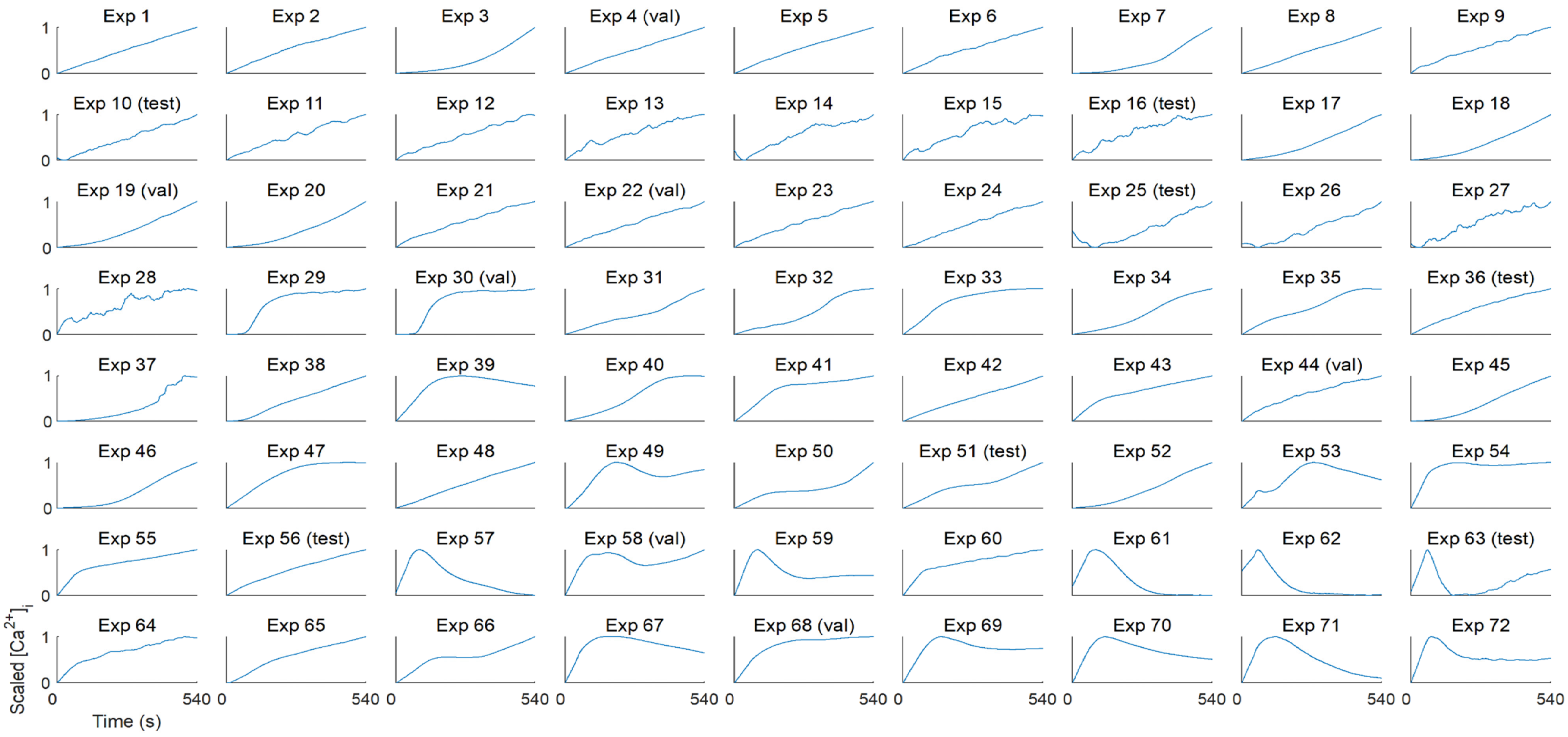

Figure S5. Scaled agonist-induced $[Ca^{2+}]_i$ time traces of Fura-2-loaded platelets from a single donor. Numbers for experiments and agonist/treatment conditions are indicated in Table 1. Raw input data in nM were curve interpolated, smoothened and linearly scaled 0-1. Time axes are from 0 to 540 s.

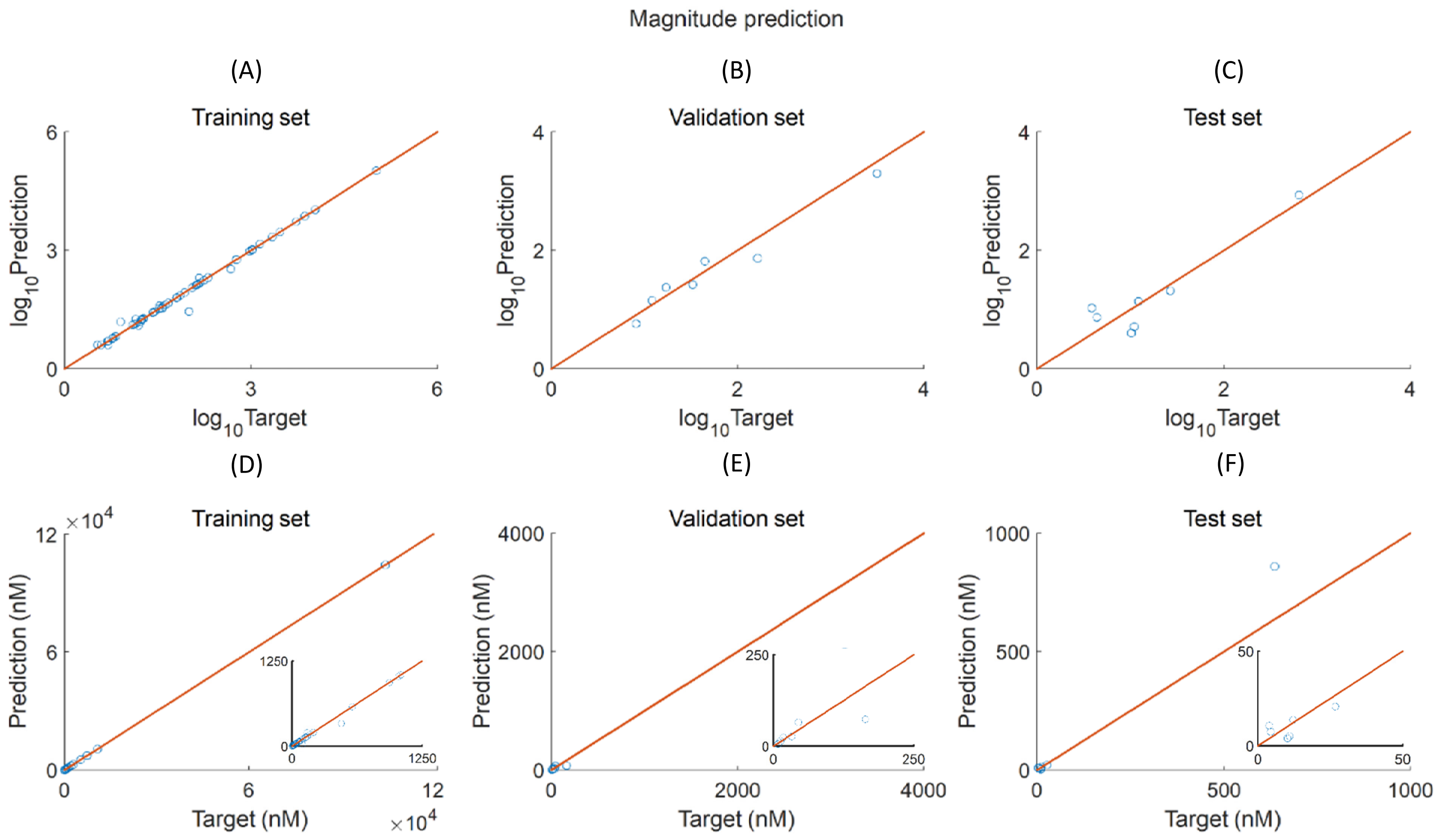

Figure S6. Magnitude prediction of agonist-induced platelet [Ca^{2+}]$_i$ responses. Shown are for time traces of the selected training set (**A, D**), validation set (**B, E**) and test set (**C, F**), the relation between the measured target levels and the predicted levels (nM). (**A-C**) Log10 scale, (**D-F**) linear scale. Note the used curve selection for validation set (Exp. 4,19,22,30,44,58,68), test (Expt. 10,16,25,36,51,56,63), and training set (all the rest). Red lines represent diagonals.

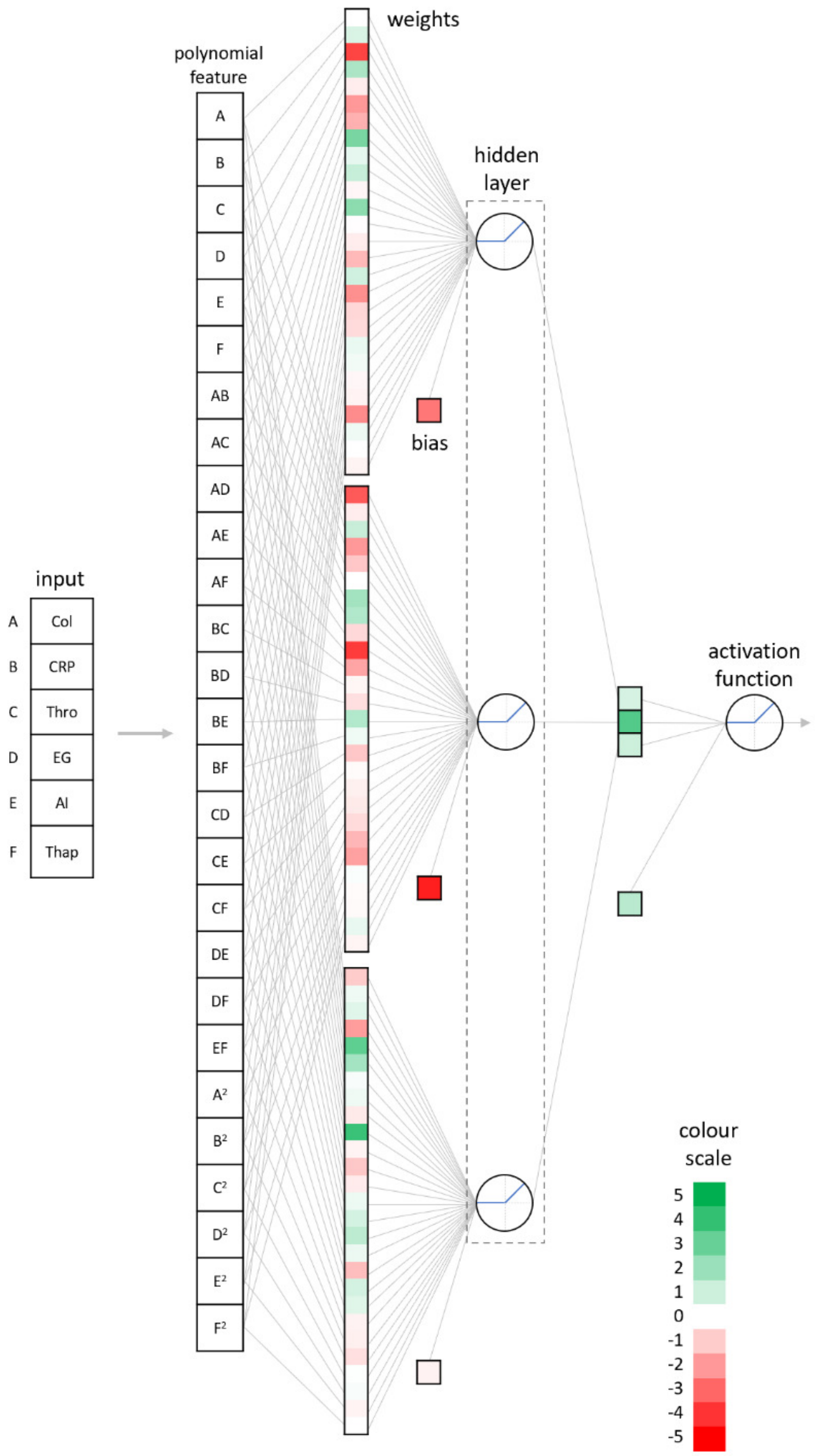

Figure S7. Parameter composition of polynomial multilayer perceptron (MLP) network associated with each node. Note the 27 combinations obtained from 6 input variables. Relative weights of per node are displayed in colour scale.

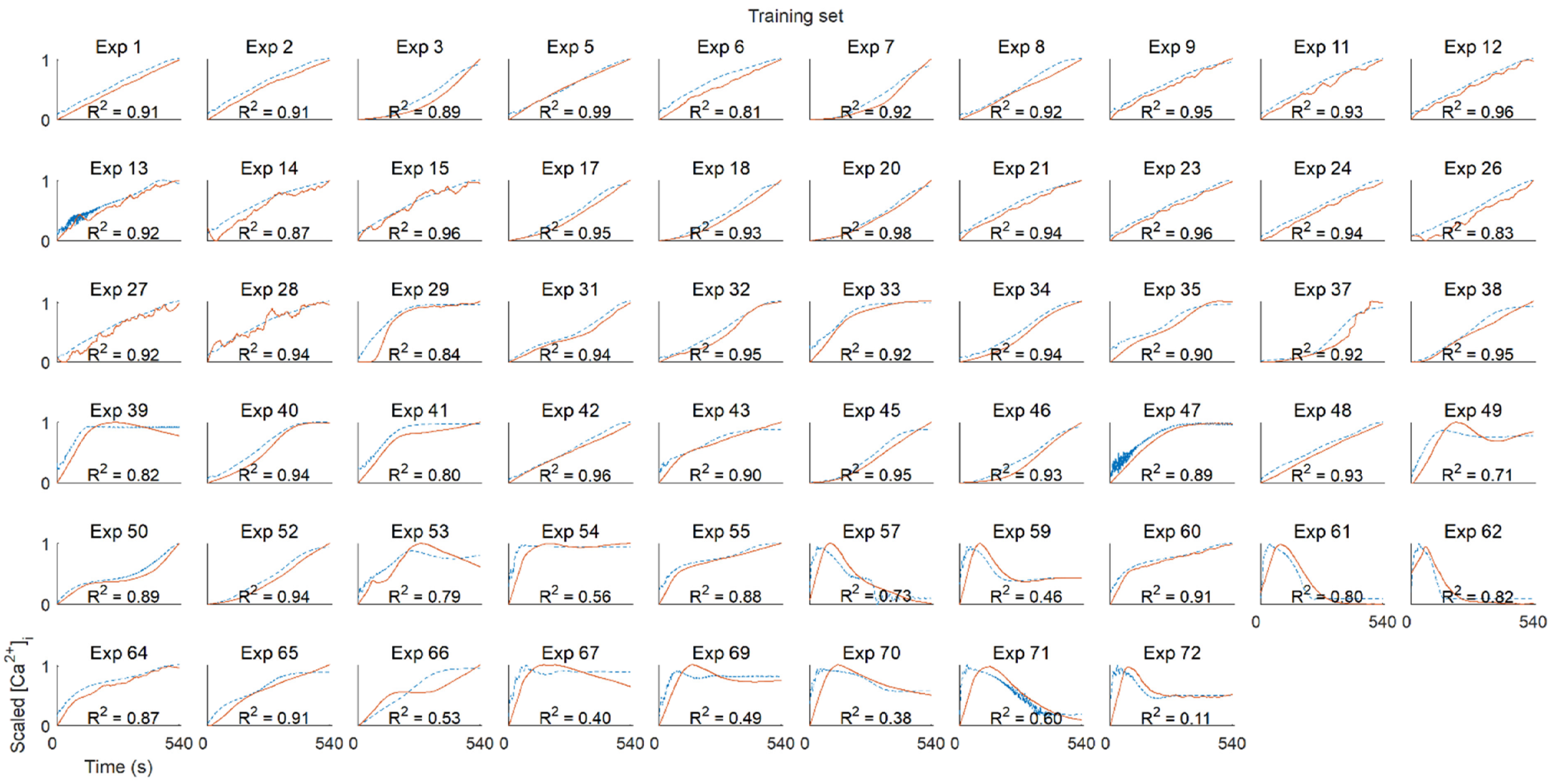

Figure S8. Trend prediction with NARX of agonist-induced platelet [Ca^{2+}]$_i$ responses in the training set. Numbers for experiments and agonist/treatment conditions are indicated in Table 1. Vertical axes represent scaled responses from 0 to 1. Horizontal axes represent measurement time from 0 to 540 s.

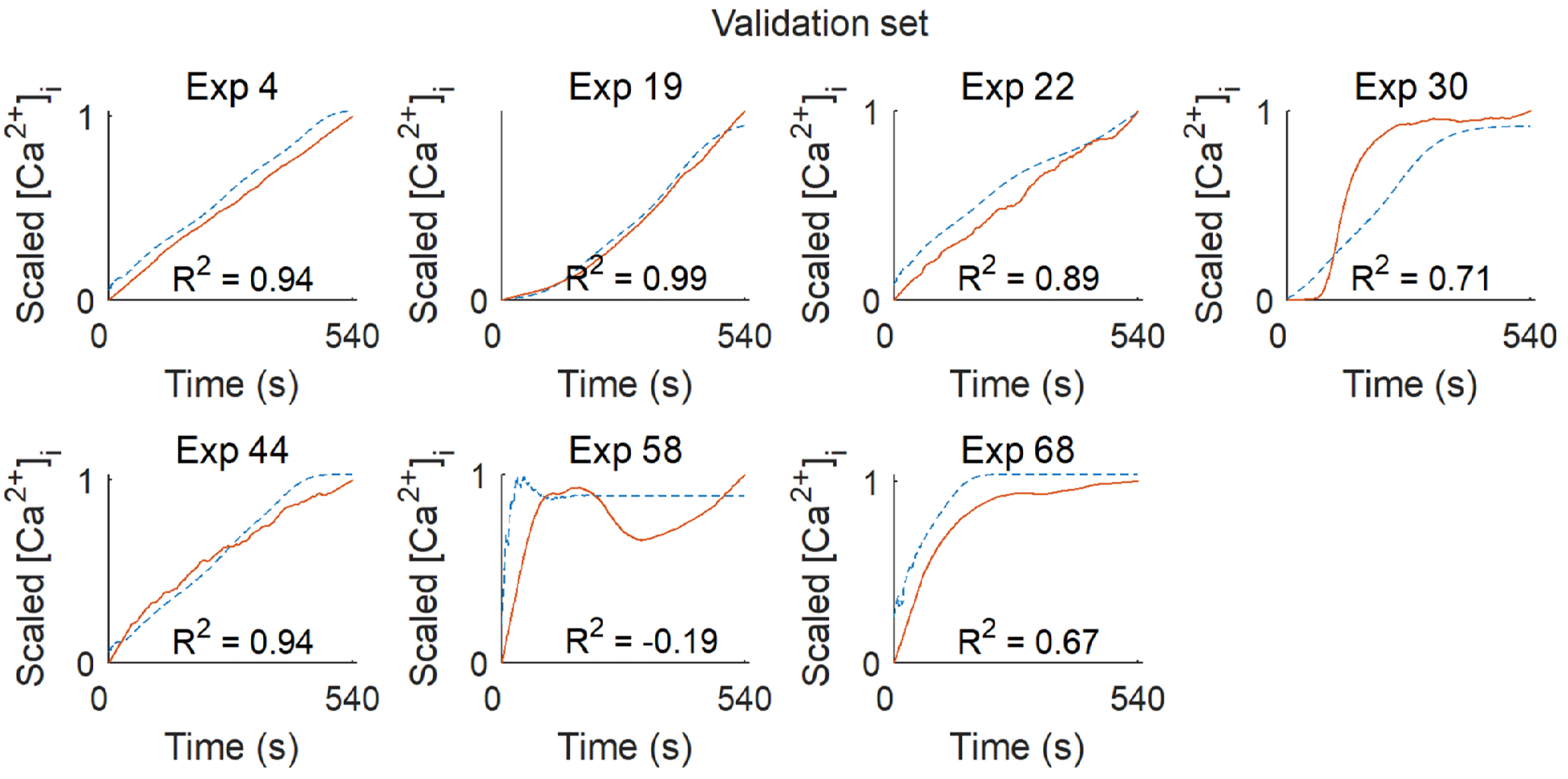

Figure S9. Trend prediction of agonist-induced platelet $[Ca^{2+}]_i$ responses in the validation set. Experimental conditions were numbered as in Table 1. Vertical axes represent scaled responses from 0 to 1. Horizontal axes represent experimental time from 0 to 540 s.

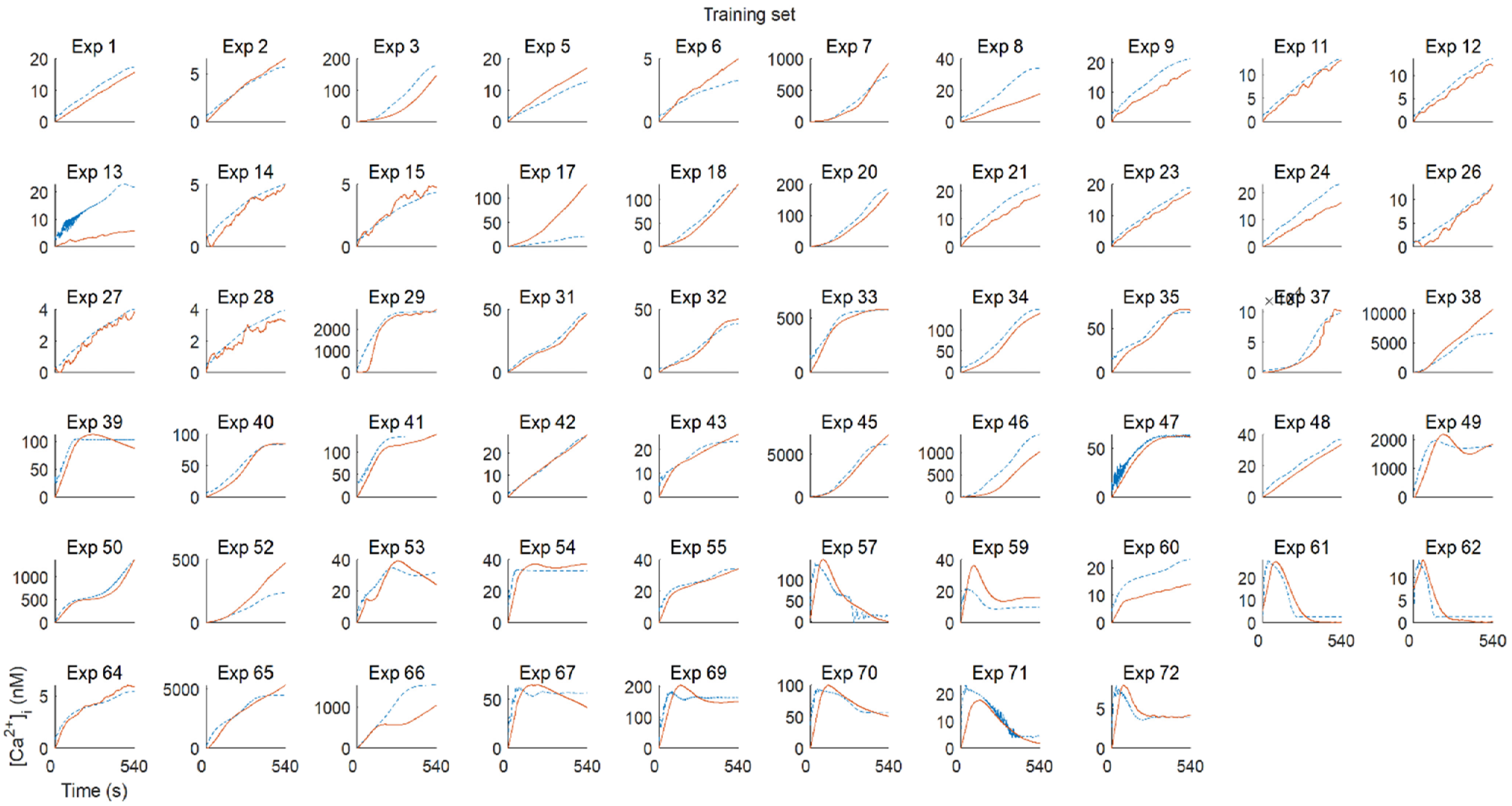

Figure S10. Magnitude and trend prediction of platelet [Ca^{2+}]$_i$ responses in training set. The predicted curves resulted from the combination of magnitude and trend predictions. Numbers for experiments and agonist/treatment conditions are indicated in Table 1. Panels indicate nanomolar [Ca^{2+}]$_i$ levels versus time (0 to 540 s).

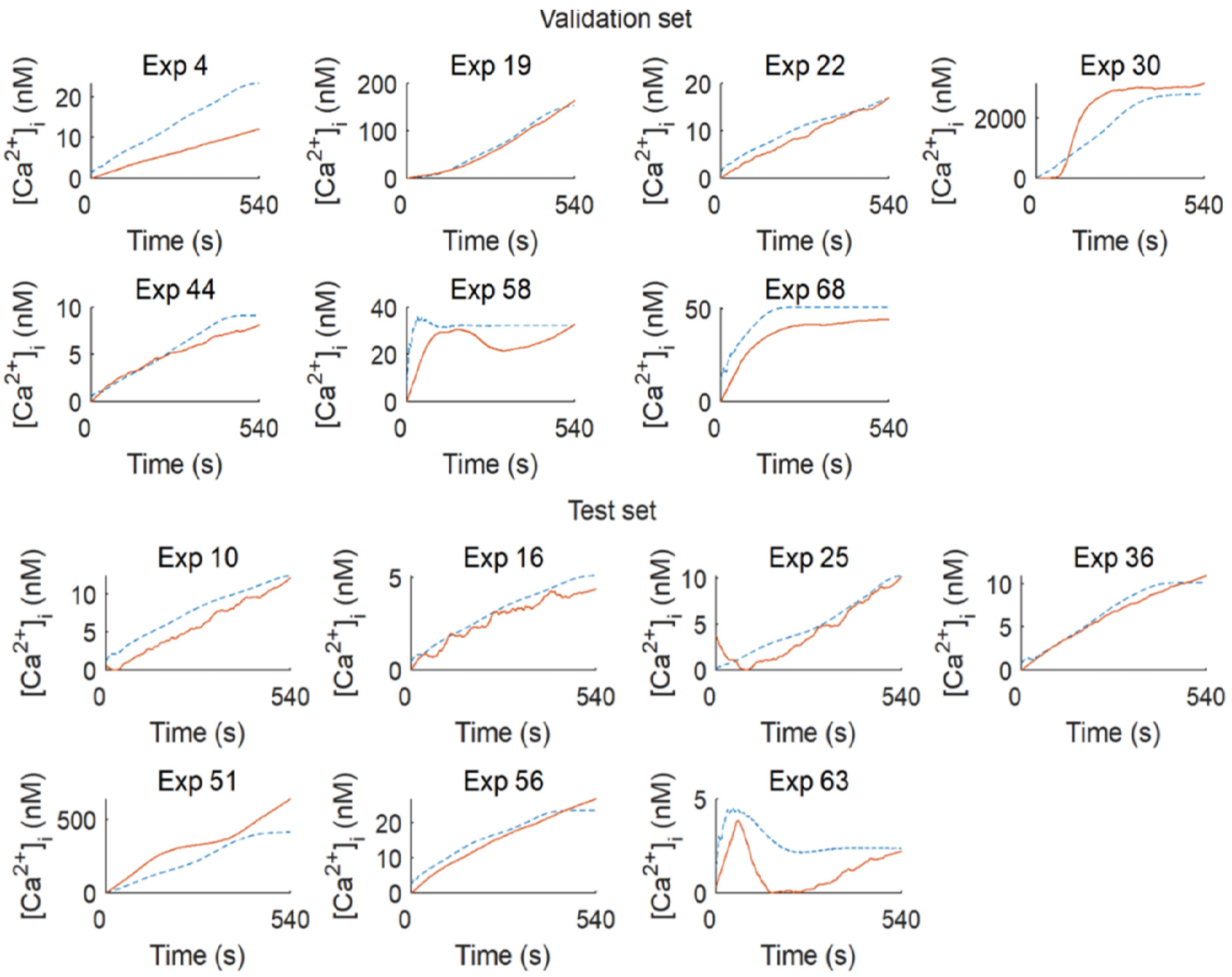

Figure S11. Combined prediction of platelet $[Ca^{2+}]_i$ responses with magnitude and trend predictions. Indicated are results from validation set (**A**) and from test set (**B**). The results from 2 models were combined (magnitude and trend prediction). Numbers for experiments and agonist/treatment conditions are indicated in Table 1. Vertical axes indicate $[Ca^{2+}]_i$ levels in nM, and horizontal axes the time.

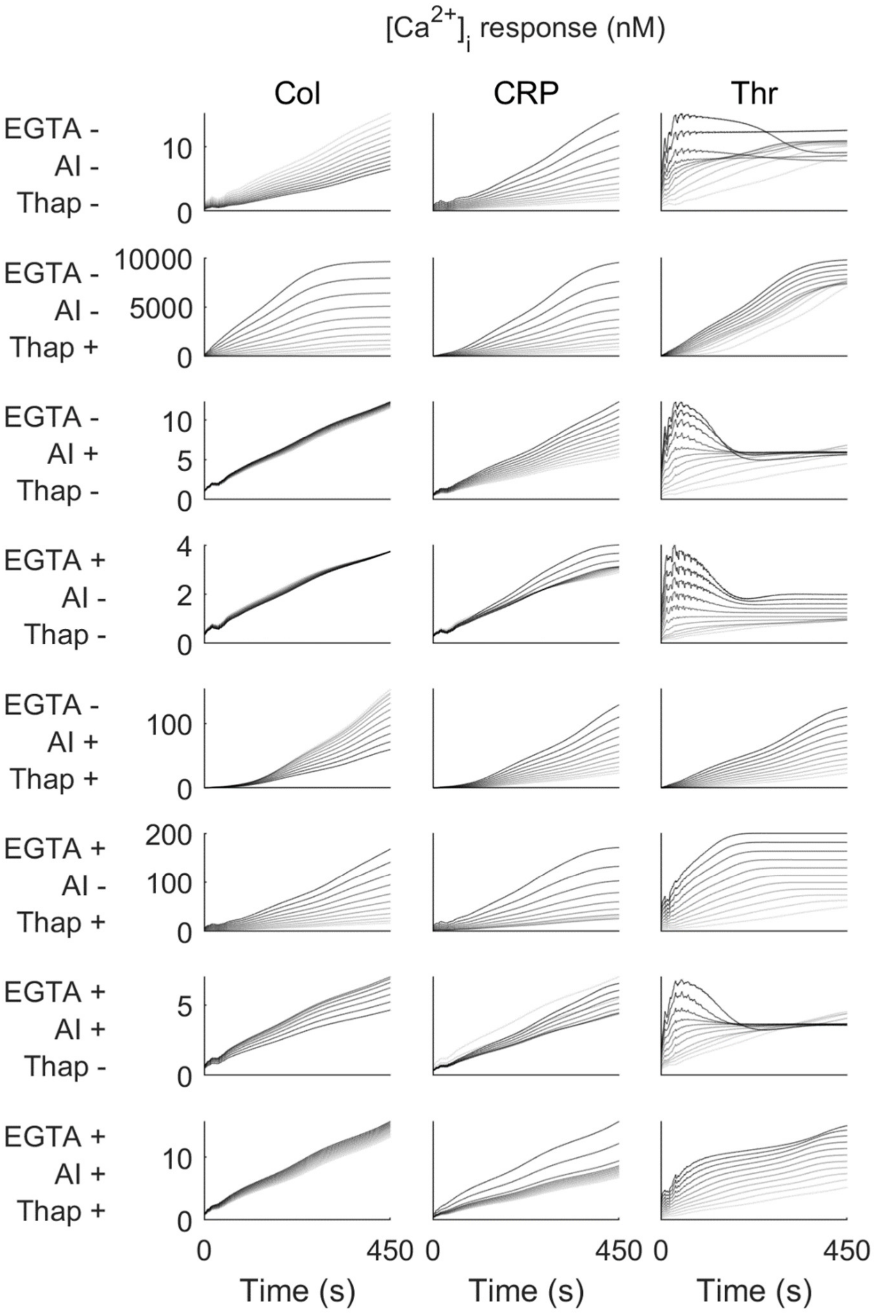

Figure S12. Variation of trend prediction of $[Ca^{2+}]_i$ time curves with increasing agonist concentrations. Panels indicate prediction efficacy per agonist concentration. Lightest grey lines represent basal levels, while darker lines represent a curve prediction due to an increment of ligand by 1% from basal level to 10% of the maximum concentration used in the training set. Columns show conditions with different agonists (collagen, Col), CRP or thrombin (Thr). Rows represent different inhibitor conditions: + or - indicates presence or not. From top to bottom: EGTA, apyrase plus indomethacin (AI), and thapsigargin (Thap). Shown are unscaled levels of $[Ca^{2+}]_i$ (nM); for scaled data, see Figure 5.

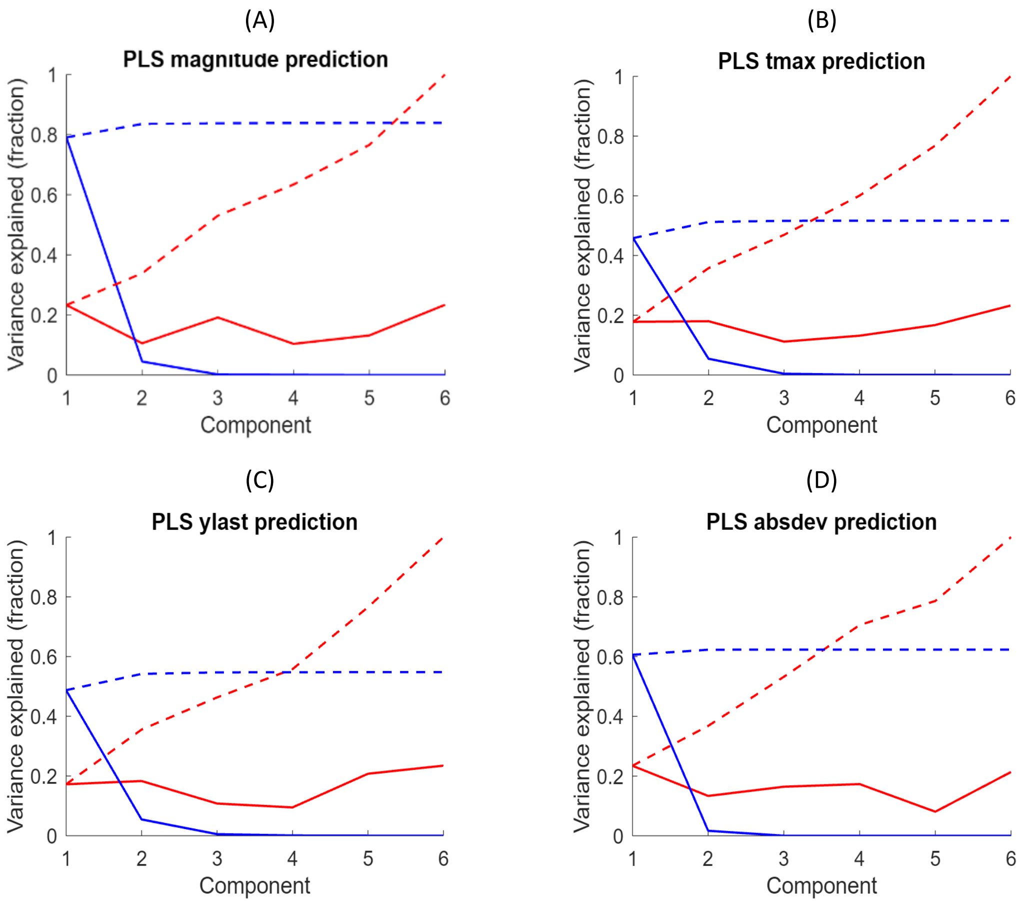

Figure S13. Variance in PLS regression model explained per component. PLS regression analysis was performed for prediction of curve *magnitude* (**A**), *tmax* (**B**), *ylast* (**C**) and *absdev* (**D**). Plots show per principal component the fraction of explained variance of the dataset. Red lines indicate the explained variance of input (experimental condition); blue lines show explained variance of the target (scalar curve characteristic). Dashed lines display cumulative sums with increasing components. Note that only 2 components contributed to the the target variance. For the included experimental variables, see Figure 6.

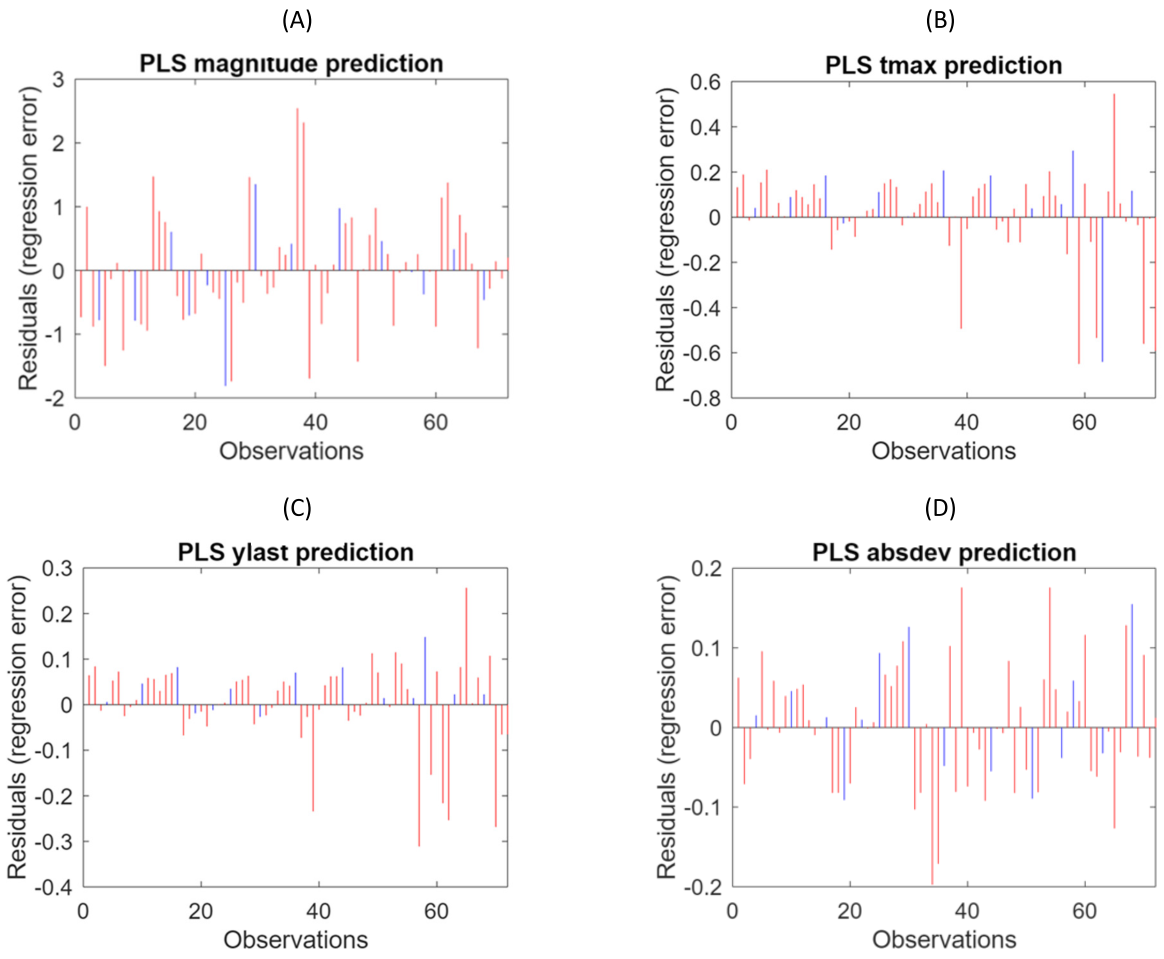

Figure S14. Residual deviations of the PLS regression model. PLS regression analysis was performed for prediction of curve *magnitude* (**A**), *tmax* (**B**), *ylast* (**C**) and *absdev* (**D**). Indicated are regression errors upon increasing observations in the training set (red bars) and test set (blue bars). Regression errors were defined as the actual minus the predicted value. Positive errors indicate an underestimate of the explained variance.

► **Figure S15. Modelling of drug effects affecting SOCE by PLS regression analysis of scaled $[Ca^{2+}]_i$ curves.** Fura-2-loaded platelets were triggered with CRP (10 µg/mL) or thrombin (10 nM) with EGTA/$CaCl_2$ or thapsigargin (, as indicated. (**A**) Shown are 16 sets of representative nM $[Ca^{2+}]_i$ curves over 540 s with vehicle medium (control) or in the presence of drug 2APB (30 µM), i.e. an established Ca^{2+} entry blocker. Curves are representative for n=3-5 subjects, see Refs. 15 and 28. (**B**) Results of PLS regression analysis for prediction of the scalar curve characteristics: *magnitude* (**a**), *tmax* (**b**), *ylast* (**c**) and *absdev* (**d**). Plots show for the first two principal components loadings of the included variables, similarly to Figure 6: AI (yes/no), collagen (Col, yes/no), CRP (yes/no), thrombin (Thro, yes/no), thapsigargin (Thap, yes/no) EGTA (yes or no = $CaCl_2$). Indicated per row: predictions based on curve characteristics without drug (**i**, upper row), with drug (**ii**, middle row), combined with drug (yes/no) as additional variable (**iii**, lower row). Indicated in colors are the contributions per variable. Note the highly similar loadings in components 1 and 2 for the variables "EGTA" and "Drug" (dotted red line).

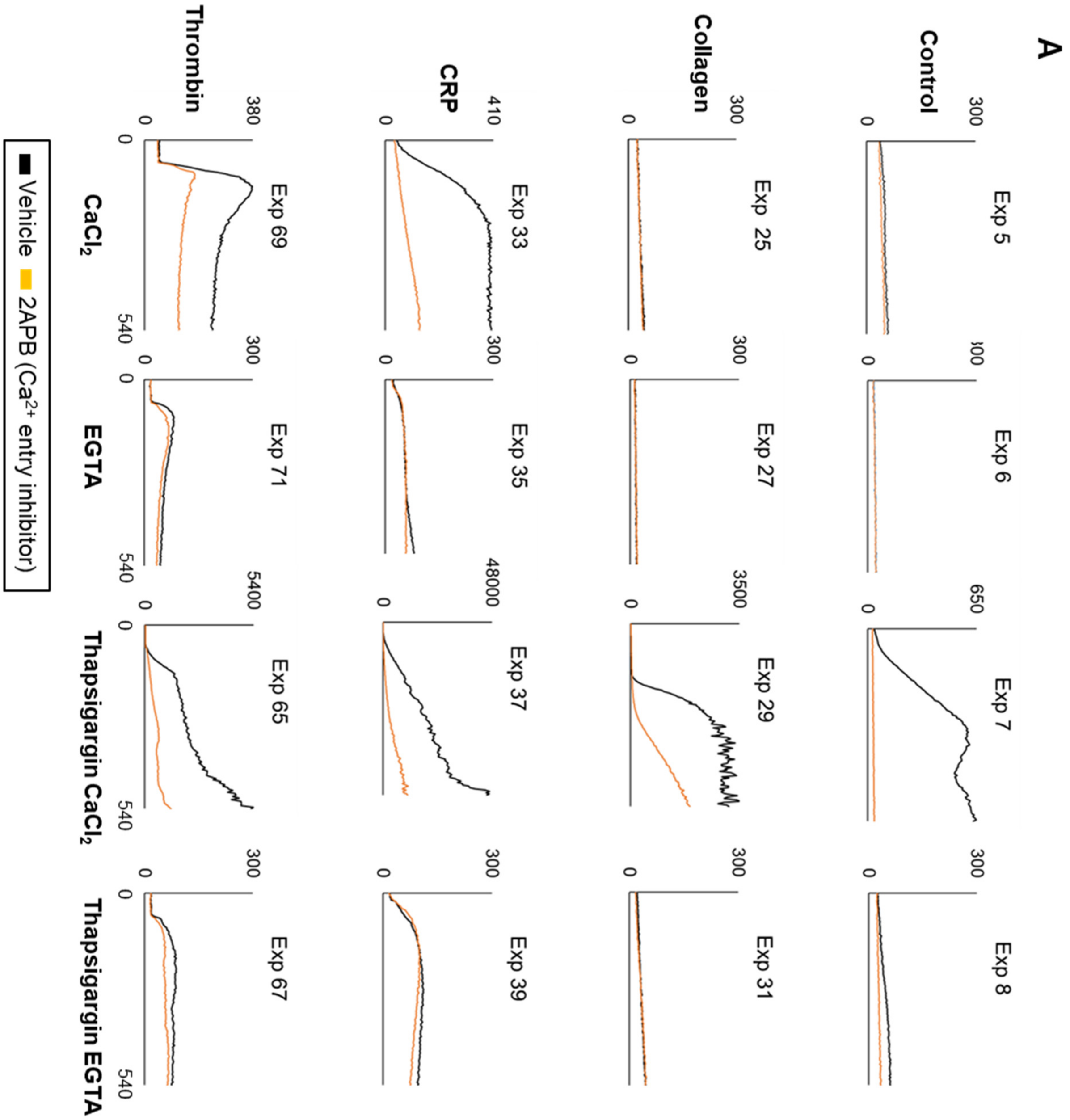
A
Control
Collagen
CRP
Thrombin
Exp 5
Exp 6
Exp 7
Exp 8
Exp 25
Exp 27
Exp 29
Exp 31
Exp 33
Exp 35
Exp 37
Exp 39
Exp 69
Exp 71
Exp 65
Exp 67
300
650
3500
410
48000
380
5400
0
540
CaCl$_2$
EGTA
Thapsigargin CaCl$_2$
Thapsigargin EGTA
Vehicle
2APB (Ca^{2+} entry inhibitor)

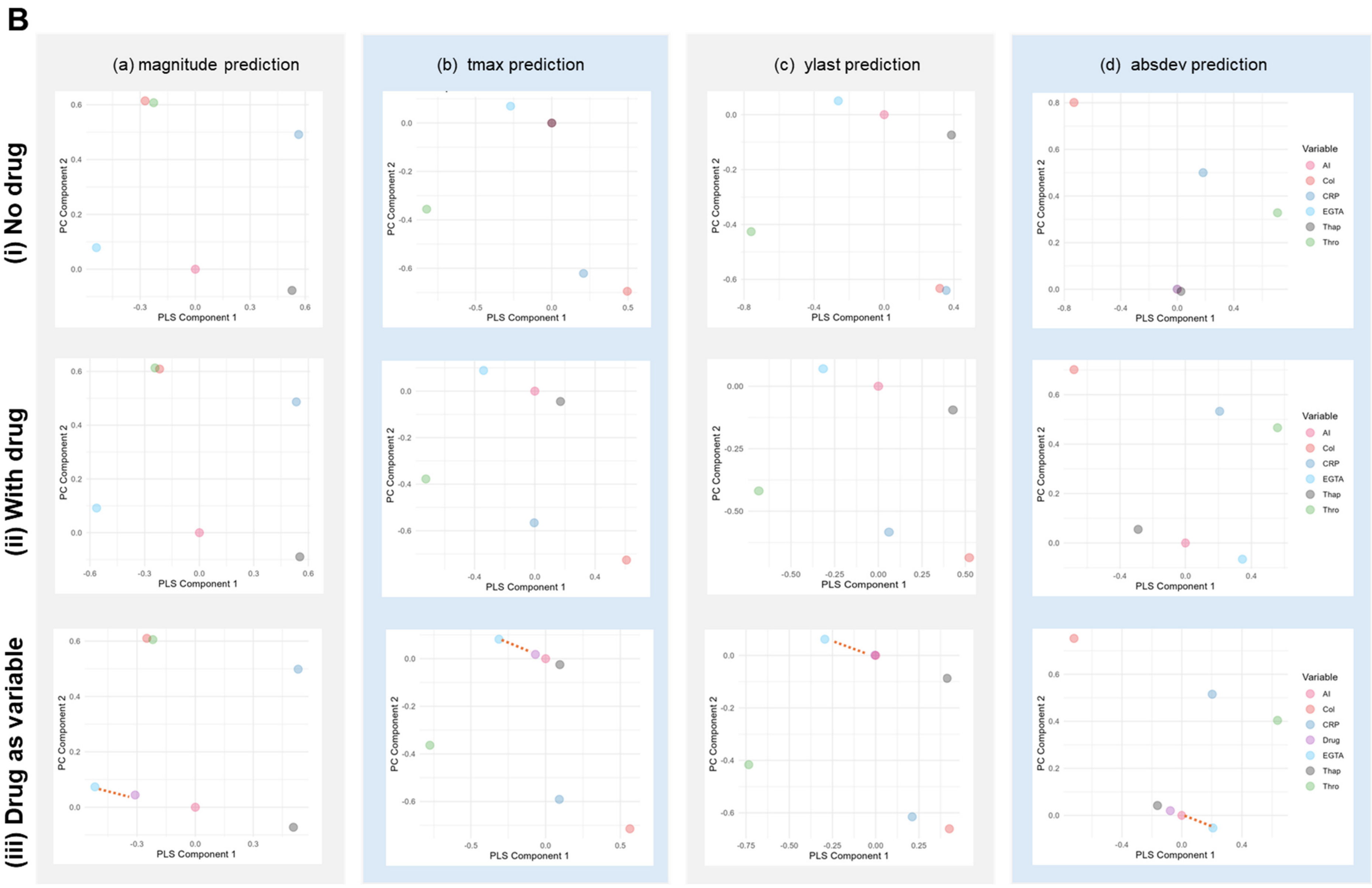
B
(a) magnitude prediction
(b) tmax prediction
(c) ylast prediction
(d) absdev prediction
(i) No drug
(ii) With drug
(iii) Drug as variable
PC Component 2
PLS Component 1
Variable
AI
Col
CRP
Drug
EGTA
Thap
Thro
